# Reduced voltage losses yield 10% efficient fullerene free organic solar cells with >1 V open circuit voltages†
†Electronic supplementary information (ESI) available. See DOI: 10.1039/c6ee02598f



**DOI:** 10.1039/c6ee02598f

**Published:** 2016-11-09

**Authors:** D. Baran, T. Kirchartz, S. Wheeler, S. Dimitrov, M. Abdelsamie, J. Gorman, R. S. Ashraf, S. Holliday, A. Wadsworth, N. Gasparini, P. Kaienburg, H. Yan, A. Amassian, C. J. Brabec, J. R. Durrant, I. McCulloch

**Affiliations:** a Department of Chemistry and Centre for Plastic Electronics , Imperial College London , London , SW7 2AZ , UK . Email: d.baran@imperial.ac.uk; b IEK5-Photovoltaics , Forschungszentrum Jülich , 52425 Jülich , Germany . Email: t.kirchartz@fz-juelich.de; c Faculty of Engineering and CENIDE , University of Duisburg-Essen , Carl-Benz-Straße 199 , 47057 Duisburg , Germany; d King Abdullah University of Science and Technology (KAUST) , KSC , Thuwal 23955-6900 , Saudi Arabia; e Institute of Materials for Electronics and Energy Technology (I-MEET) , Friedrich-Alexander-University Erlangen-Nuremberg , Erlangen , Germany; f Department of Chemistry and Hong Kong Branch of Chinese National Engineering Research Center for Tissue Restoration & Reconstruction , Hong Kong University of Science and Technology , Clear Water Bay , Kowloon , Hong Kong , China

## Abstract

Optimization of the energy levels at the donor–acceptor interface of organic solar cells has driven their efficiencies to above 10%. However, further improvements towards efficiencies comparable with inorganic solar cells remain challenging because of high recombination losses, which empirically limit the open-circuit voltage (*V*_oc_) to typically less than 1 V. Here we show that this empirical limit can be overcome using non-fullerene acceptors blended with the low band gap polymer PffBT4T-2DT leading to efficiencies approaching 10% (9.95%). We achieve *V*_oc_ up to 1.12 V, which corresponds to a loss of only *E*_g_/*q* – *V*_oc_ = 0.5 ± 0.01 V between the optical bandgap *E*_g_ of the polymer and *V*_oc_. This high *V*_oc_ is shown to be associated with the achievement of remarkably low non-geminate and non-radiative recombination losses in these devices. Suppression of non-radiative recombination implies high external electroluminescence quantum efficiencies which are orders of magnitude higher than those of equivalent devices employing fullerene acceptors. Using the balance between reduced recombination losses and good photocurrent generation efficiencies achieved experimentally as a baseline for simulations of the efficiency potential of organic solar cells, we estimate that efficiencies of up to 20% are achievable if band gaps and fill factors are further optimized.

## Abstract 2

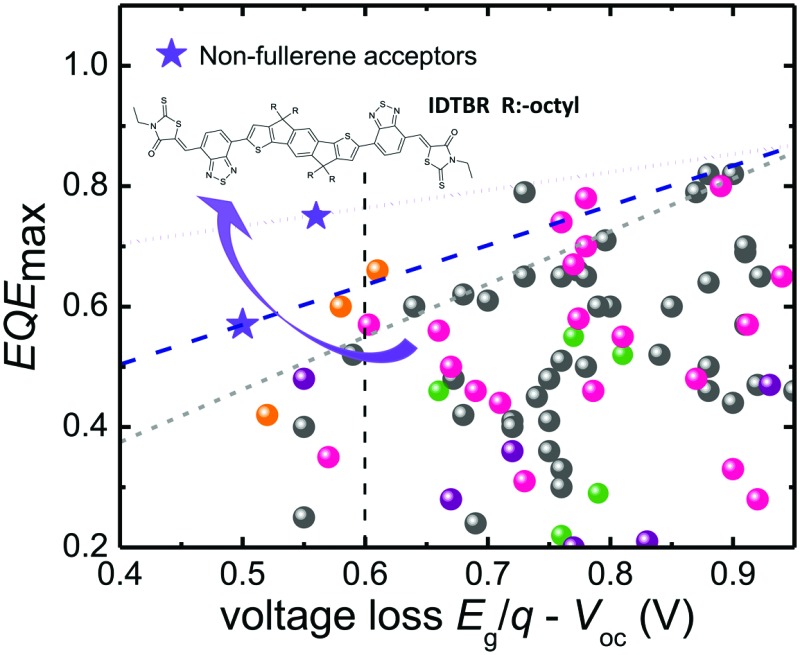
Non-fullerene acceptors with optimized energy levels enable 10% efficient solar cells with reduced voltage losses <0.6 V.

Broader context

Recently, organic solar cells have reached power conversion efficiencies (PCE) up to 12%. However, the compromise between voltage loss and external quantum efficiency (EQE) still limits the PCE of these devices for further improvements with devices providing open circuit voltages (*V*_oc_) > 1 V. In this report, we present a guideline for reducing the compromise between voltage loss and EQE by using small-molecule acceptors and achieve PCEs up to 10% without the need for fullerene acceptors. By replacing fullerenes with different small molecule acceptors, we were able to achieve efficiencies up to 10% with *V*_oc_ values >1 V (up to 1.12 V) and EQE values approaching 76%. These improvements are achieved by suppressing recombination losses in the donor:acceptor blends and optimizing the nano-structure for efficient charge separation at the interface. Our simulations predict that these suppressed losses with high EQE values can yield up to 20% organic solar cells provided further optimization of fill factor and band gap is possible in the future.

One of the main criteria for highly efficient solar cells is the combination of high photocurrent densities (*J*_sc_) with high open-circuit voltages (*V*_oc_). In the case of organic solar cells, the heterojunction between an electron donating and an electron accepting material is required for efficient exciton dissociation leading to a compromise between these two parameters. In order to achieve the highest possible photocurrent some of the achievable *V*_oc_ has to be sacrificed.^1–3^ Thus, a substantial amount of work on organic photovoltaics has focused on minimizing the voltage loss required for exciton dissociation and to achieve the best possible compromise between photocurrent and photovoltage.^4–9^ Fig. 1 demonstrates the empirical consequences of this compromise showing that the smaller the voltage difference between the band gap *E*_g_ (divided by the elementary charge *q*) and the actual *V*_oc_, the smaller will be the efficiency of photocurrent generation represented by the maximum external quantum efficiency (EQE_max_) (see Table S1, ESI† for details). Below a voltage loss of about *E*_g_/*q* – *V*_oc_ ≈ 0.6 V, there are hardly any reports in the literature of reasonable photocurrents and EQE_max_ values above 50%, with a few notable recent exceptions including using non-fullerene acceptors.^5,6,10^ Although some of these reports focused on minimizing the voltage loss *E*_g_/*q* – *V*_oc_ whilst keeping EQE as high as possible, the *V*_oc_ of the corresponding devices remained still below 1 V. Furthermore, the values for *J*_sc_ and fill factor (FF) were comparatively lower than those of their fullerene containing counterparts.^11–14^ Thus, there seems to be an empirical limit both to the *V*_oc_ and the voltage loss and it is difficult to overcome this limit even using NFA alternatives.^4–6,12^ So far, *V*_oc_ values exceeding 1 V in organic solar cells have been achieved with very few acceptors.^9,15,16^ The relatively large voltage losses obtained to date with organic solar cells have been related to the observation of high non-radiative recombination losses if compared to many inorganic and hybrid solar cell materials.^17^ Materials such as GaAs or metal-halide perovskites, for instance, have achieved voltage losses as low as 0.22 V, due to the lower non-radiative recombination losses in these cells, as demonstrated for example by the relatively high electroluminescence yields (EQE_EL_) reported for these devices.^18–20^ The EQE_EL_ for organic solar cells is usually very weak (in the range of 10^–6^ to 10^–4^%) due to weakly absorbing charge transfer (CT) states. As such, a key challenge for organic solar cells is increased the EQE_EL_ which is viable with the suppression of such non-radiative recombination losses.^19,21,22^ Finding a donor:acceptor combination which simultaneously has minimized energetic offset for charge separation (Δ*E*_CS_) and high EQE_EL_ (>10^–4^) with suppressed non-radiative recombination losses (<0.3 V) along with a favourable nanoscale morphology for reduced non-geminate recombination to give high EQE and fill factors (FFs), is still a challenge in OPV. Small molecule acceptors (SMA) have emerged as strong alternatives to fullerene derivatives in organic solar cells in the last couple of years.^5,6^ Their high absorption coefficients and tunable absorption spectra in the visible range enables enhanced photon harvesting and higher LUMO energy levels which contribute to photocurrent and photovoltage in bulk-heterojunction (BHJ) devices. Although high efficiencies are reported for fullerene free solar cells, their *V*_oc_ values are still limited <1 V along with high EQE values.^6^

**Fig. 1:**
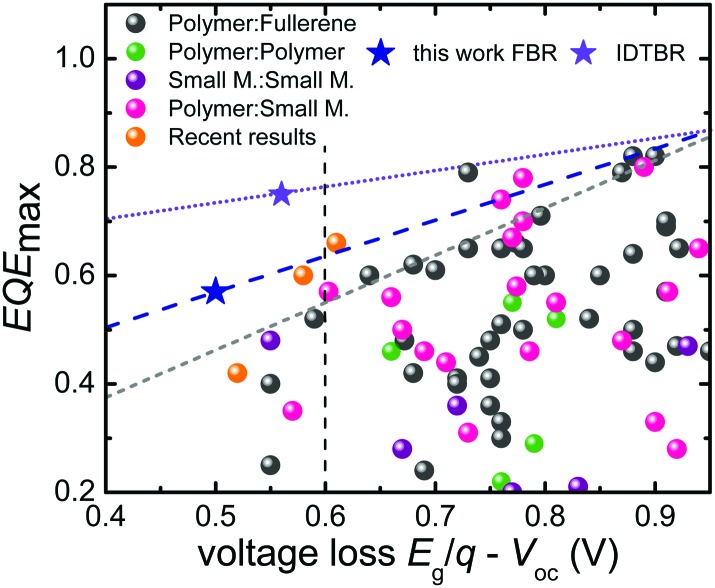
Comparison of the EQE_max_ and the voltage loss between *E*_g_/*q* and the *V*_oc_ for different types of organic solar cells. The grey line indicates the empirical limit for the maximum EQE possible for a given voltage loss given by Li *et al.* ^4^ The blue and violet lines are determined using the result in the current work. Traditionally, there are no cases of EQE_max_ > 0.7 with a voltage loss <0.6 V. Recent results presented here as well as the results from Kawashima and Yan *et al.* ^5,10^ show that non-fullerene acceptors are promising candidates to overcome this barrier.

Here we show that the empirical limits on the origin of 1 *V*_oc_ and voltage-loss can be overcome by selecting four different donor:NFA combinations using fluorene, indenofluorene and indacenodithiophene based NFAs (see ESI† for details) and a low band gap polymer PffBT4T-2DT, resulting in power conversion efficiencies (PCEs) as high as 7.8% and 9.95% when FBR and IDTBR molecules are used as acceptors, respectively. These values are achieved without any pre- or post-treatment methods or additives. The most remarkable aspects of the devices are high *V*_oc_ = 1.12 V (*E*_g_/*q* – *V*_oc_ ≈ 0.5 V), with high EQE (>55%) and *J*_sc_ (11.5 mA cm^–2^) for PffBT4T-2DT:FBR and 1.07 V *V*_oc_ and 75% EQE (*J*_sc_ = 15 mA cm^–2^) for PffBT4T-2DT:IDTBR devices with EQE_EL_ values up to 10^–3^%,which is the largest EQE_EL_ for organic bulk-heterojunction solar cells achieved so far. The results show that NFA devices have an order of magnitude higher electroluminescence quantum yield compared to the fullerene analogue device and to the rest of the NFA solar cells resulting in low non-radiative losses (Δ*V*_oc_) which maximize the *V*_oc_.^10^ The crystalline nano-morphology of the PffBT4T-2DT:FBR blend results in suppressed non-geminate recombination and non-Langevin behaviour and efficient charge transport whilst maintaining efficient exciton dissociation with very small charge separation energy (Δ*E*_CS_).^15^ A *V*_oc_ greater than 1.1 V at an optical band gap around 1.6 eV is promising and comparable to the *V*_oc_ values of best Pb-halide perovskites published in the literature.^20,23–26^ Thus, these results show that organic bulk heterojunction (BHJ) solar cells have the potential to achieve photo-voltages compatible with solar cells that do not require a heterojunction for exciton dissociation.

## Results

**Fig. 2:**
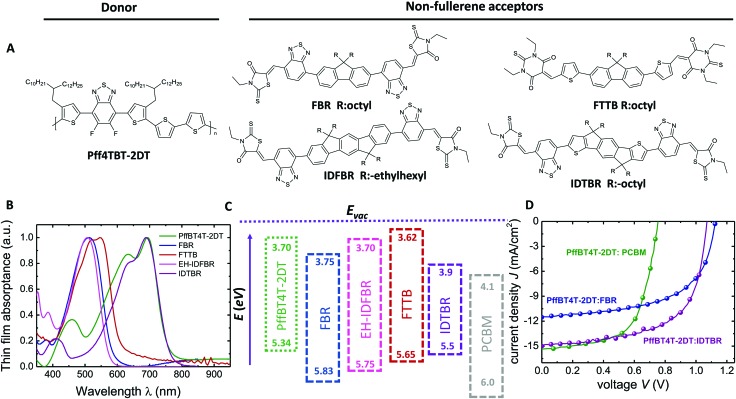
Chemical structures, optical data and device performances of materials used. (A) Chemical structures of PffBT4T-2DT and acceptors used in this study. (B) Normalized UV-vis absorption spectra of neat films. (C) Energy level diagram for the donor and acceptor materials obtained from film CV measurements. EA of PffBT4T-2DT and IP of acceptors are estimated from the optical bandgaps of the materials. (D) Current–voltage curves of PffBT4T-2DT:FBR devices compared with PffBT4T-2DT:PCBM under illumination of 100 mW cm^–2^.

The molecular structures of PffBT4T-2DT and the acceptor molecules are illustrated in Fig. 2A and their complementary film absorption properties are shown in Fig. 2B. Recently, FBR was reported as a visible absorbing alternative acceptor to PCBM and used for solar cells with P3HT.^27^ Although the efficiency was improved (4.1%) relative to PCBM (3.5%) due to an increased *V*_oc_, overlapping absorption profiles of P3HT (530 nm) and FBR (510 nm) limited the amount of photocurrent at longer wavelengths and initiated the search for an alternative donor material whose absorption and energy levels are more complementary to FBR than P3HT (Table S2, ESI†). Two different non-fullerene acceptors (IDFBR and FTTB) have been designed and synthesized which absorb similar to FBR and therefore have complementary absorption spectra to many low band gap polymers (the synthetic details of the IDFBR and FTTB can be found in the ESI†). The extended conjugation of the aromatic system in IDFBR leads to a slight raise both in highest occupied molecular orbital (HOMO) and lowest unoccupied molecular orbital (LUMO) levels compared to FBR (Fig. 2C). FTTB exhibits a higher extinction coefficient than FBR (Fig. S1a, ESI†) due to replacing the rhodanine end group with a stronger dye chromophore – thiobarbituric acid – which effectively contributes to the photon harvesting.^28^ The low band gap acceptor IDTBR has also shown to work nicely with P3HT; however, the high lying HOMO energy level of P3HT limited the *V*_oc_ below 1 V in these devices.^29^

Cyclic voltammetry (CV) is utilized to determine the electron affinity (EA) and ionization potential (IP) of individual materials and estimate the energy offset driving charge separation (Δ*E*_CS_), defined as the difference between the energy of the singlet exciton energy and that of charge separated polarons, where Δ*E*_CS_ = ((IP_D_ – EA_A_) – *E*_S1_) (Table S1, ESI†). We note this energy offset is analogous to the lowest unoccupied molecular orbital (LUMO) level offset Δ*E*_LUMO_ between donor and acceptor, but includes the effect of the exciton binding energy in lowering each materials optical bandgap relative to its electronic bandgap.^30^ The IP of PffBT4T-2DT and EA of acceptors FBR, IDFBR and FTTB are calculated as 5.34 eV, 3.75 eV, 3.70 eV, 3.62 eV, respectively from the film CVs and the corresponding EA and IP values were estimated using the *E*_g_ values of the neat films (Fig. 2C). Using these data, a polaron pair energy of 1.64 ± 0.02 eV is calculated for the PffBT4T-2DT:FBR blend. The optical band gap (exciton energy) of PffBT4T-2DT is calculated from the onset of the absorption spectrum as *E*_g,opt_ = 1.61 ± 0.02 eV. Whilst both measurements have significant systematic errors, they do indicate a remarkably small (near zero) energy offset driving charge separation, Δ*E*_CS_ = 30 ± 40 meV (Fig. S1B and C, ESI†).^31,32^ A similar conclusion is reached from our estimated LUMO energy offset for this blend, Δ*E*_LUMO_ ≈ 50 meV, significantly smaller than that reported previously for other efficient donor/acceptor blends.

The photovoltaic properties of the PffBT4T-2DT:FBR and PffBT4T-2DT:IDTBR blends are investigated by fabricating devices with a ITO/ZnO/active layer (*d* ≈ 120 nm)/MoO_*x*_/Ag architecture using different donor:acceptor (D/A) ratios without any additional annealing or processing additives. Current–voltage (*J*–*V*) characteristics of the PffBT4T-2DT:FBR and PffBT4T-2DT:IDTBR cells with different D/A ratios exhibit the best performance at a 1 : 1 D/A ratio spin coated from chlorobenzene (Fig. S2a, ESI†). The key photovoltaic parameters and the maximum EQE values are presented in Table 1 and Table S3 (ESI†) (for the D/A variations). PffBT4T-2DT:IDTBR devices with a 1 : 1 D/A ratio spin coated from chlorobenzene give even higher efficiencies of 9.95% with high photocurrent (15 mA cm^–2^) and *V*_oc_ of 1.07 V. Both PffBT4T-2DT:FBR and PffBT4T-2DT:IDTBR outperform PffBT4T-2DT:PC_71_BM devices, prepared following a previously reported recipe using the processing additive 1,8-diiodooctane (DIO), in efficiency and much more prominently in *V*_oc_ (see Fig. 2D and Table 1).^31^ In addition to the three devices shown in Fig. 2D we tried two more NFA with PffBT4T-2DT and achieved *V*_oc_s above 1 V but not high efficiencies (see Table S2, ESI† for information on the NFAs). While PffBT4T-2DT:IDTBR has the highest efficiency among all the PffBT4T-2DT-based organic solar cells investigated in this study, we are most interested in the origin of the high open-circuit voltages.^33,34^ Therefore, we studied PffBT4T-2DT:FBR, the blend with the highest *V*_oc_, in more detail in the following.

**Table 1:** Photovoltaic performances of the solar cells based on PffBT4T-2DT and various acceptors (FBR, IDTBR and PCBM) under standard AM 1.5G illumination

PffBT4T-2DT:FBR	*J*_sc_ ^*b*^ (mA cm^–2^)	*V*_oc_ (V)	FF (%)	PCE_ave_ ^*c*^ (%)	EQE@*λ*_max_ (%)
1 : 1^*a*^	11.5 (±0.20)	1.12 (±0.01)	61 (±0.2)	7.8 (±0.20)	57 (±0.2)
PffBT4T-2DT : IDTBR (1 : 1)	15.0 (±0.20)	1.07 (±0.01)	62 (±0.2)	9.95 (±0.20)	76 (±0.2)
PffBT4T-2DT : PCBM (1 : 2) (3% DIO)	16.0 (±0.20)	0.76 (±0.01)	62 (±0.2)	7.5 (±0.3)	70 (±0.2)

^*a*^Surface of ZnO is modified with washing the layer with the solvent of zinc acetate.

^*b*^Short circuit density measured from *J*–*V* measurements.

^*c*^PCE_ave_: average power conversion efficiency with standard deviation from 12 devices.

## Discussion

**Fig. 3:**
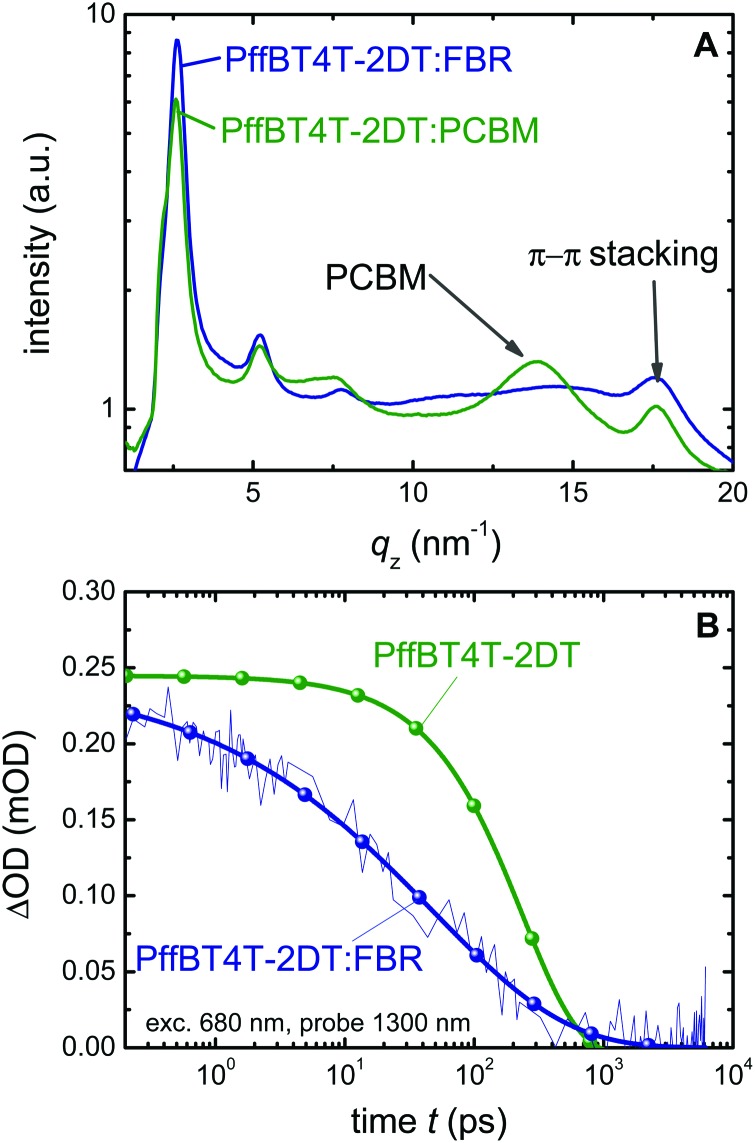
Microstructural and time resolved analysis of PffBT4T-2DT based films. (A) Integrated scattering intensity of the as cast PffBT4T-2DT:FBR and PffBT4T-2DT:PCBM films from grazing incident wide angle X-ray scattering (GIWAXS) measurements. (B) Transient absorption spectra of neat PffBT4T-2DT and PffBT4T-2DT:FBR films excited at 680 nm and probed at 1300 nm. The blend has much shorter exciton lifetime (48 ps) than the neat polymer (237 ps).

To investigate the effect of the acceptor on the crystallinity of PffBT4T-2DT, we perform grazing incidence wide angle X-ray scattering measurements (GIWAXS) measurements and the integrated scattered intensities of the PffBT4T-2DT:FBR and PffBT4T-2DT:PCBM films are demonstrated in Fig. 3A. 2D plots of GIWAXS pattern are given in the ESI† (Fig. S3). Both PffBT4T-2DT:FBR and PffBT4T-2DT:PCBM show (*h*00) reflections corresponding to lamellar stacking distance (*q*) of PffBT4T-2DT crystals which are centred around *q* ∼ 2.6 nm^–1^ (100), 5.2 nm^–1^ (200) and 7.6 nm^–1^ (300). The diffraction from (*h*00) reflections with an arc-like shape is indicating the presence of a random distribution of the crystals, although we observe slightly more dominance in the out-of-plane direction. Diffraction from π–π stacking (010 reflection) is observed for both samples centred around *q* ∼ 17.6 nm^–1^ with its azimuth distribution reveals a strong tendency for face-on orientation. This is confirmed by a pole figure of the π–π stacking reflection, shown in Fig. S5c (ESI†). To gain quantitative insight into the structural changes, the integrated intensity of lamellar stacking (100) peak was fitted to a Gaussian profile to calculate the crystalline correlation length (CCL), a parameter related to the size of a crystallite.^35^ We observe slightly higher CCL of Pff4TBT-2DT crystallites in Pff4TBT-2DT:FBR (CCL ∼ 13.5 nm) as compared to PCBM analogue (CCL ∼ 10.8 nm) indicating larger crystallites size for Pff4TBT-2DT:FBR.

### Charge generation and recombination

The fact that organic solar cells do not usually achieve high photocurrents for low voltage losses (*E*_g_/*q* – *V*_oc_) is often due to an increase in exciton decay to the ground state and/or geminate recombination of bound polaron pairs, typically associated with a reduction in the Δ*E*_CS_ at the donor–acceptor heterojunction.^31^ Δ*E*_CS_ is extremely low with this materials system and it is therefore remarkable that photocurrent generation is found to be efficient. In order to study the exciton dynamics of the neat polymer and PffBT4T-2DT:FBR blend in more detail, we use microsecond (μs) (Fig. S4A, ESI†) and femtosecond (fs)-transient absorption spectroscopy (TAS) as shown in Fig. 3B. The samples are excited at 680 nm, *i.e.* close to the absorption maximum of the polymer, and probed at 1300 nm, which represents the polymer singlet exciton decay (Fig. S4B, ESI†). The neat polymer transient is used as a reference and allows us to estimate a polymer singlet exciton lifetime of *τ*_r_ = 237 ps. The singlet exciton lifetime in the blend is about *τ*_eff_ = 48 ps, which we assume to be due to a combination of exciton decay and exciton dissociation (*τ*_d_) given by *τ*_d_ = (*τ*_eff_^–1^ – *τ*_r_^–1^)^–1^. From this we conclude that about 20% of the excitons created on the polymer in the blend are lost due to recombination and about 80% dissociate. The 20% exciton losses can be attributed to either the coarse and crystalline structure of the PffBT4T-2DT:FBR film, due the highly crystalline texture of PffBT4T-2DT or the low energy offset driving exciton separation Δ*E*_CS_. The nanomorphology of the active layer usually determines the exciton dissociation efficiency and charge transport properties in the device. Pure and crystalline phases may result in increased losses due to exciton decay to ground, but has been shown to result in slower recombination losses, thus facilitating charge collection and enabling higher voltage generation.^36^ Unusually, the lifetime of PffBT4T-2DT is relatively long compared to similar highly crystalline-small bandgap polymers (such as DPP based polymers).^37^ This allows us to have a coarse and crystalline blend microstructure while still having relatively efficient exciton harvesting (charge generation) at the PffBT4T-2DT:FBR interface and good charge collection properties simultaneously. These TAS data were further supported by PL quenching data (Fig. S4C, ESI†), which indicate that illumination at both the donor and acceptor absorption maxima results in PL quenching of about 85%, consistent with our ultrafast TAS data. This modest PLQ is similar to that observed for annealed P3HT:PCBM blends, which is a well investigated robust microstructure in OPV but suffers from large Δ*E*_CS_ between P3HT and PCBM. The moderate PL quenching of PffBT4T-2DT:FBR film indicate a coarse nano-morphology which is strongly related to the crystalline nature of PffBT4T-2DT previously reported and discussed above.^38^

### Charge transport

**Fig. 4:**
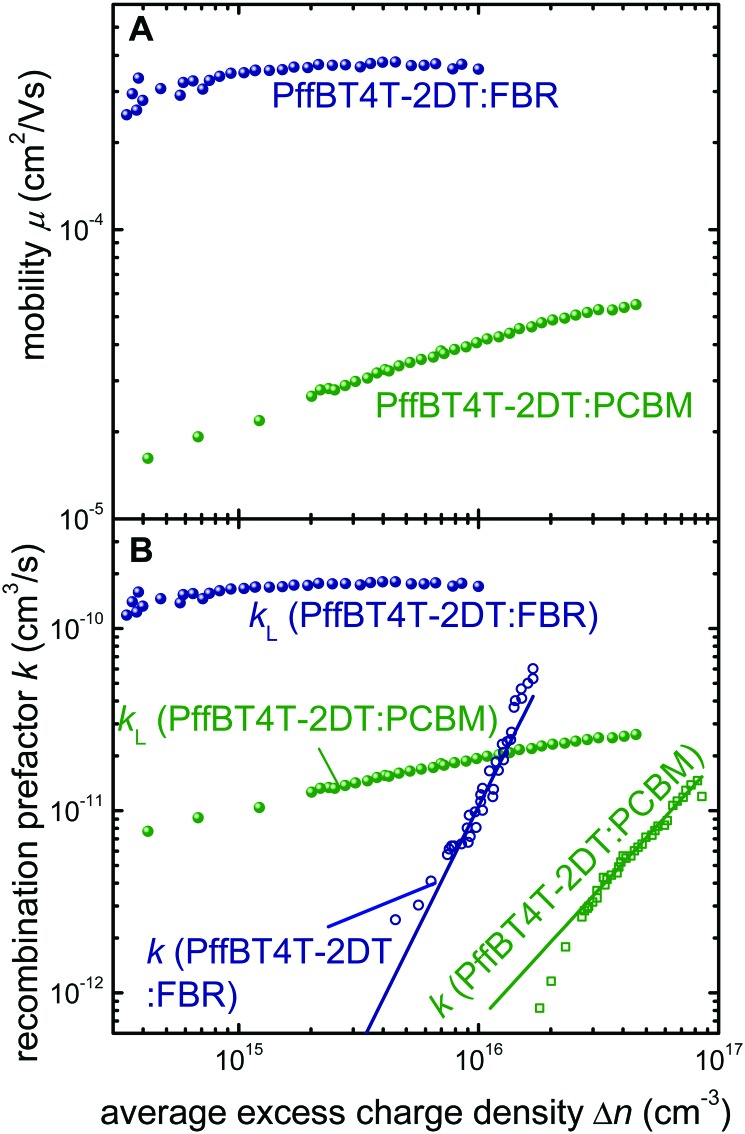
Analysis of recombination and transport in PffBT4T-2DT based devices. (A) Effective device mobility *μ*_CE_ as a function of charge carrier density, measured by charge extraction at short circuit for PffBT4T-2DT:PCBM (green) and PffBT4T-2DT:FBR (blue). (B) Langevin recombination rate constant (circles) and the measured bimolecular recombination rate (open squares) measured from TPV and CE at open circuit (ESI†).

In order to study the anticipated positive consequences of the coarse microstructure, we study the charge transport properties using the charge extraction at short circuit method.^39,40^ Fig. 4A shows the determined mobility as a function of charge density for the two different acceptor blends. We find that the mobility of PffBT4T-2DT:FBR is substantially higher than that of PffBT4T-2DT:PCBM devices at all charge carrier densities. High charge carrier mobilities are beneficial for efficient charge extraction but have to be combined with long charge carrier lifetimes.^41–43^ Thus, we studied transient photovoltage (TPV) lifetimes of the two blends in combination with ideality factors from dark *JV* curves and *V*_oc_ *vs.* light intensity measurements. From the latter measurements, we can conclude that recombination with low ideality factors, *i.e.* bimolecular recombination between quasi free charge carriers, is suppressed in both blends with ideality factors being mostly between 1.5 and 2 (Fig. S5, ESI†). Such ideality factors require either very broad band tails or deep traps.^44^ The ideality factor of the PffBT4T-2DT:FBR decreases at higher light open-circuit voltages which is a typical feature observed when surface recombination is dominant.^45^ Recombination in the bulk, however, is due to high ideality factors observed at low voltages, a situation that is uncommon in organic photovoltaics.^46^ Typically, many of the intimately mixed blends have ideality factors ≈1 as they are limited by bimolecular recombination between free charge carriers or charge carriers trapped in shallow defect or tail states, whilst higher ideality factors have been reported mostly for materials with coarse or crystalline microstructures such as P3HT:PCBM.^45^ The observation of higher ideality factors in P3HT:PCBM is evidence for reduced bimolecular recombination rather than increased trap concentration which implies that high ideality factors might be (counterintuitively) beneficial for device performance.^46^ The high ideality factors measured here are consistent with the TAS and PL quenching data that suggest a coarse microstructure for both PffBT4T-2DT:FBR and PffBT4T-2DT:PCBM blends. In order to find out whether the high ideality factors correlate with slow or fast recombination kinetics, we studied the recombination coefficients (*k*) as a function of average excess charge carrier density (Δ*n*). Fig. 4B shows the *k* in comparison with the recombination coefficients *k* = *qμ*_CE_/*ε*_0_ *ε*_r_ derived from the Langevin theory.^47^ Here *q* is the elementary charge, *μ*_CE_ is the mobility as determined from charge extraction^39,40^ and as displayed in Fig. 4A (ESI†), *ε*_0_ is the vacuum permittivity and *ε*_r_ is the relative permittivity. From Fig. 4B, it becomes clear that while PffBT4T-2DT:FBR has higher recombination coefficients than PffBT4T-2DT:PCBM, the values are still substantially below those predicted by Langevin theory. Thus, PffBT4T-2DT:FBR has suppressed bimolecular recombination between free carriers which helps establishing a higher photovoltage relative to PffBT4T-2DT:PCBM.

### The origin of the high *V*_oc_

**Fig. 5:**
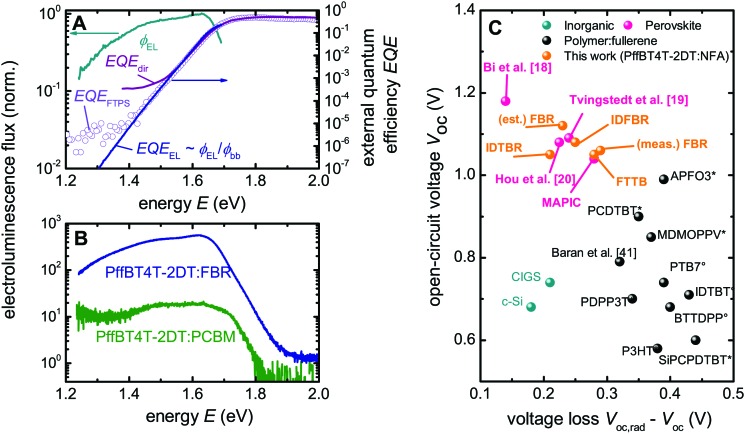
Calculation of the voltage loss PffBT4T-2DT:FBR and comparison with other solar cells. (A) The EQE (solid line) determined from the EL, the directly measured quantum efficiency (purple line) and FTPS (open circles) used to determine the *V*_oc,rad_ (1.35 V) of the PffBT4t-2DT:FBR device and the voltage loss due to non-radiative recombination (Δ*V*_oc_). (B) Electroluminescence emission spectra comparison of PffBT4t-2DT:PCBM and PffBT4t-2DT:FBR cells at a given current density (150 mA cm^–2^) using Si-detector. EL emission spectrum of PffBT4t-2DT:IDTBR using InGaAs detector can be found in ESI† Fig. 6. (C) Comparison of the relation between *V*_oc_ and Δ*V*_oc,nr_ for different photovoltaic technologies including this work with non-fullerene acceptors. * Denotes the devices made with PC_60_BM and ° is for the devices made with PC_70_BM, using the data from ref. 17. The estimated voltage loss for FBR (est. FBR) is calculated by assuming the best FBR devices have similar *V*_oc,rad_ as average FBR devices. For further information about the different devices, see Table S3 (ESI†).

Using transient photovoltage, we have shown above that the recombination kinetics of the studied blends are slow compared to the prediction from Langevin theory, which is one ingredient for the low voltage losses. In the following we want to study the high *V*_oc_ observed for Pff4TBT-2DT:FBR blends more closely by comparing it with the radiative and the Shockley–Queisser limit for the *V*_oc_, respectively. The Shockley–Queisser (SQ) limit for the *V*_oc_ is determined using detailed balance arguments between absorption and emission of photons and is calculated using a step-function, *i.e.*, absorptance is one above the *E*_g_ (1.61 eV in our case) and zero below it. The idea of the SQ limit is that radiative recombination is the only non-avoidable recombination mechanism and therefore provides a lower limit for recombination at a given carrier concentration and an upper limit for the open-circuit voltage. The Shockley–Queisser limit *V*_oc,SQ_ for this band gap is about 1.35 ± 0.02 V, with the error depending on the exact method of calculating the *E*_g_.^19^ The radiative limit, *V*_oc,rad_, is calculated in the same way as *V*_oc,SQ_, however instead of a step-function like absorptance, the real quantum efficiency is measured using sensitive Fourier transform photocurrent spectroscopy (FTPS) and electroluminescence spectroscopy (EL) as shown in Fig. 5A (Fig. S6, ESI†). For organic solar cells, *V*_oc,SQ_ is typically substantially higher than *V*_oc,rad_ due to the energy loss that is due to the emission from the CT state (which dominates *V*_oc,rad_) being shifted towards lower energies as compared to the absorption onset (which determines *V*_oc,SQ_). In the case of PffBT4T-2DT:FBR, both are nearly equal. Thus, the donor–acceptor interface is not a limiting factor for the *V*_oc_ loss, which is similar to the case of inorganic solar cells, perovskite solar cells or some low bandgap polymer:fullerene blends like PDPP3T:PCBM.^17–19^ Thus, the only remaining voltage loss is due to non-radiative recombination (Δ*V*_oc,nr_), which is the voltage difference between *V*_oc,rad_, where all the recombination is radiative, and the measured *V*_oc_.^41^ Note that this voltage loss Δ*V*_oc,nr_ should not be confused with the voltage difference between *E*_g_/*q* and *V*_oc_, which was displayed in Fig. 1 and which cannot easily be assigned to individual loss mechanisms.^48^

The loss Δ*V*_oc,nr_ will be minimized where the radiative recombination current leading to measurable electroluminescence is maximized relative to the total recombination current as shown in Fig. 5B for PffBT4T-2DT:FBR devices compared to PffBT4T-2DT:PCBM devices. Fig. 5C gives a summary of the values of the loss Δ*V*_oc,nr_ for different organic and inorganic solar cells. We obtain a remarkably small non-radiative Δ*V*_oc,nr_ loss of only 290 mV for PffBT4T-2DT:FBR devices (*V*_oc_ = 1.06 V of the device measured in EL) and 270 mV for PffBT4T-2DT:IDTBR, which is smaller than for PffBT4T-2DT:PCBM (330 mV) devices (Table 1). The voltage loss Δ*V*_oc,nr_ is calculated to be only 230 mV if we take the best *V*_oc_ (1.12 V) and assume that the *V*_oc,rad_ doesn’t change between different PffBT4T-2DT:FBR devices. Both values for NFAs are better than any other value previously reported for organic solar cells and comparable to those of the best perovskite solar cells. If we take the *V*_oc_ (1.06 V) of the device that was used for the EL measurement to obtain Δ*V*_oc,nr_ = 290 mV which is still lower than most previously reported values for organic solar cells. Fig. 5C illustrates that the values of Δ*V*_oc,nr_ achieved for NFA systems are all below 0.3 V, and are much smaller than those typically reported for organic solar cells. In addition, these values are comparable to those of the best perovskite solar cells in literature. All these losses result in very high EQE_EL_ in the range of 10^–3^ for FBR and IDTBR devices, respectively and the results are summarized in Table 2. These values are the largest electroluminescence yield reported for organic solar cells so far. The EQE_EL_ is described as a route to achieve high *V*_oc_ for organic solar cells.^3^ However, weakly absorbing CT states result in very low yields in the range of 10^–4^ to 10^–6^ in these devices limit the *V*_oc_. Using NFAs as a route to increase EL emission yield result in lower voltage losses in PffBT4T:FBR devices compared to PCBM analogue (Fig. S6E, ESI†). Thus, using alternative non-fullerene acceptors in OPV which shows that the intrinsic disadvantages of bulk heterojunctions can be largely overcome leading to devices with high luminescence despite the large internal interface area.^18,19^

**Table 2:** Parameters measured and calculated for quantifying the non-radiative recombination losses for all of the devices studied in this work

	PffBT4T-2DT:FBR (av. cell)	PffBT4T-2DT:IDTBR	PffBT4T-2DT:PC_71_BM
*J*_0,rad_ (A cm^–2^) calc.^*b*^	2.7 × 10^–25^	9.4 × 10^–25^	4.97 × 10^–18^
*V*_oc,rad_ (V) calc.^*b*^	1.35	1.32	1.09
*V*_oc_ (V) measured^*a*^	1.12 (1.06)	1.05	0.76
Δ*V*_oc,nr_ (V)^*c*^	0.23 (0.29)	0.27	0.33
EQE_EL_ (%)	1 × 10^–2^ (1 × 10^–3^)	3 × 10^–3^	1 × 10^–4^
PCE (%)^*d*^	7.80	9.95	7.50

^*a*^ *J*_sc_ and *V*_oc_ were taken from the *J*–*V* curves.

^*b*^ *J*_0,rad_ and *V*_oc,rad_ were calculated from EL and FTPS measurements.

^*c*^Δ*V*_oc_ from *V*_oc,rad_ – *V*_oc_.

^*d*^See ESI for further details about photovoltaic parameters.

**Fig. 6:**
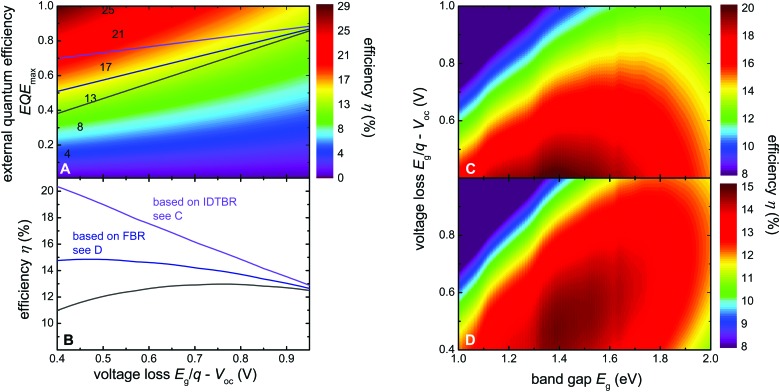
Efficiency potential simulated as a function of voltage loss and maximum EQE. (A) Maximum possible efficiency as a function of the EQE_max_ and the voltage loss. The three lines correspond to the lines in Fig. 1 and represent the empirical trend given by ref. 4 and the trend given by the present result (blue and violet lines). (B) The efficiency *vs.* voltage loss that can be obtained on the three lines. Notably, the new result shifts the point of maximum efficiencies to very low voltage losses (<0.5 V), while the earlier empirical line led to a maximum efficiency at voltage losses in the range of 0.7 to 0.8 V which corresponds to current record cells like PffBT4T-2OD:PC_71_BM.^36^ (C) Efficiency as a function of band gap and voltage loss assuming an EQE_max_ given by the violet lines in (A) and (B) and the highest possible FF for a solar cell without any resistive losses. (D) The same as (C) but for the blue line.

Finally, we perform simulations on the efficiency potential of single junction organic solar cells as a function of EQE_max_ and voltage loss (*E*_g_/*q* – *V*_oc_) as illustrated in Fig. 6A. In addition to the voltage loss *E*_g_/*q* – *V*_oc_ and EQE_max_, the optical band gap *E*_g,opt_ is a third variable that is required to directly calculate the efficiency of a solar cell by assuming that the quantum efficiency is a step function that has the value EQE_max_ for energies above the *E*_g,opt_ and zero for energies below. The FF could be considered as an optimization criterion but there is a clear definition of the maximum possible FF as a function of *V*_oc_ that can be used for this purpose.^49,50^ Thus, for each combination of *E*_g_/*q* – *V*_oc_ and EQE_max_ we varied the *E*_g,opt_ to obtain the highest possible efficiency. The violet, blue and grey lines in Fig. 6A and B are identical to the data from Fig. 1 and indicate the empirical upper limit from ref. 4 and the result presented herein. The change in slope between EQE_max_ and voltage loss has a tremendous effect on the obtainable device efficiency and leads to completely different optimization criteria. Current record efficiency blends with fullerenes such as PffBT4T-2OD:PC_71_BM have voltage losses of 0.8 V and exhibit efficiencies around 11%.^36^ The voltage loss of 0.8 V is close to the ideal voltage loss leading to the maximum efficiency on the grey line suggested by the literature (Fig. 6B).^4^ Thus, for previous efficiency predictions, the lower EQE values for low voltage losses over-compensated the higher *V*_oc_ such that it was not favourable to optimize polymers for low voltage losses. However, if it becomes possible to achieve higher EQEs at low voltage losses such as shown in the present paper, the violet and blue lines become more realistic approximations of the efficiency potential. While the optimum efficiency is merely shifted towards lower voltage losses for the blue line, the maximum completely disappears for the violet curve. In this scenario the efficiency enhancement from a low voltage loss dominates because the EQE_max_ dependence on voltage loss is almost flat which potentially leads to a substantial boost in efficiency. This has not yet been achieved with the current blends due to lower than optimum FFs and higher than ideal band gaps. In the past low band gap polymers with band gaps ∼1.4 eV have been introduced and efficiencies of >7% have been reached with fullerene acceptors.^51^ However, low band gap polymers have so far not been able to overcome the efficiency of polymers like PffBT4T-2OD which have band gaps of around 1.6 eV. As mentioned above for these band gaps the highest efficiencies are obtained when the voltage losses *E*_g_/*q* – *V*_oc_ = 0.8 V or higher. NFAs have shown to enable lower voltage losses with medium band gap polymers^5,6^ (as well as current work) and it might be that combinations of low band gap polymers and NFAs will lead to a further boost of efficiencies once polymer:NFA combinations are found that work well together in terms of miscibility and solubility.

In addition, we note that our current devices have fill factors in the 60% range. This implies that higher mobilities and a further reduction of recombination constants would be required to enable better charge collection for higher FF values. Based on the results of Fig. 4, it seems as if the NFAs themselves are rather increasing the mobility. However, the polymer PffBT4T-2DT which works exceptionally well with NFAs does not provide exceptional FFs so far (whether blended with fullerenes or with NFAs). Therefore it will be necessary to study the relation between electronic properties like mobility and recombination coefficient and the suitability of a polymer to work well with NFAs in the future.

If it is possible in the future to find a lower band gap polymer with high mobilities that works well with NFAs, efficiencies between 15 and 20% might be possible already from single junction organic solar cells. Based on our current understanding there is no fundamental reason why this would be impossible, but we haven’t found a polymer:NFA combination yet that would combine all these properties.

Fig. 6C and D display the efficiency data in a slightly more conventional way with the optical band gap and the voltage loss as the two parameters. The quantum efficiency EQE_max_ used for the efficiency presented in Fig. 6C and D is not constant as is typical for similar graphs found in literature^52^ but is given by the violet (C) and blue (D) line in Fig. 6A. This implies that EQE_max_ is smoothly going down towards lower voltage losses and does not drop abruptly to zero as is assumed in the original publication by Scharber.^52^

In summary, we have shown that a combination of a low band gap polymer and non-fullerene acceptor, PffBT4T-2DT:FBR, can yield remarkably high open-circuit voltage up to 1.12 V, along with high external quantum efficiencies >55% resulting in 7.8% PCE in single-junction inverted devices without any pre- or post-treatment. Furthermore, we suggest explanations on the efficient photo-induced charge transfer and high photovoltaic performance of PffBT4T-2DT:FBR blend, where there is less than 0.1 eV Δ*E*_CS_ between donor and acceptor which is much smaller than the empirical value of 0.3 eV often reported for efficient charge separation. FBR, when mixed with PffBT4T-2DT, exhibits high power conversion efficiencies due to synergistic effects of high mobilities, recombination coefficients that are substantially lower relative to the prediction from Langevin theory and in consequence extremely low voltage losses due to suppressed non-radiative recombination. Using the same polymer, an efficiency up to 10% is possible when IDTBR is used as acceptor, keeping the *V*_oc_ values above 1 V (1.07 V) with remarkably high EQE values of 75%. This work reveals that polymer:NFA blends providing low voltage losses and good luminescence yields will further boost the performance of BHJ solar cells beyond the polymer:fullerene efficiency limit by allowing *V*_oc_ values beyond 1 V with high fill factors >60% and EQE values >75%.

## Materials and methods

### Materials

The PffBT4T-2DT polymer was supplied from He Yan group synthesized according to literature procedures in ref. 36. The FBR acceptor was synthesized as previously described in ref. 27. FTTB is synthesized according to earlier reports.^16^ Indecenofluorene (IDF) is synthesized following the earlier reports.^53^ The boronic ester of ethylhexyl-IDF was coupled with 7-bromo-2,1,3-benzothiadiazole-4-carboxaldehyde *via* Suzuki coupling. The resulting intermediate was then reacted by Knoevenagel condensation with 3-ethylrhodanine to give IDFBR in 50–60% yield (see ESI† for details). All other reagents and solvents were purchased from Sigma Aldrich and used as received. PCBM is supplied from Solenne.

### Cyclic voltammetry

CV was performed with a standard three-electrode setup with a Pt-mesh counter electrode and Ag/AgCl reference electrode, calibrated against Fc/Fc^+^ using an Autolab PGSTAT101 potentiostat. Measurements at 50 mV s^–1^ were carried on ambient spin coated films (from chlorobenzene solutions at 5 mg mL^–1^) on ITO substrates with 0.1 M tetrabutylammonium hexafluorophosphate in deoxygenated acetonitrile as supporting electrolyte.

### UV-vis absorption spectra

Thin films were prepared by the same method as devices on glass substrates and spectra were recorded on a UV2600 Shimadzu UV-vis spectrometer.

### Fabrication and characterization of devices

Solar cells are fabricated for PffBT4T-2DT:FBR and PffBT4T-2DT:PC_61_BM in an inverted architecture (glass/ITO/ZnO/active layer/MoO_3_/Ag). Pre-structured indium tin oxide (ITO) substrates were cleaned by sonication in detergent, deionized water, acetone, and isopropyl alcohol for 10 minutes each. Substrates were then treated with oxygen plasma. A ZnO layer was deposited by ambient spin coating of a zinc acetate dihydrate precursor solution (2 mL 2-methoxyethanol and 60 μL ethanolamine). Devices were then annealed for 20–25 min at 150 °C, yielding layers of 30–40 nm. Active layers of PffT2T-2DT:FBR with different ratios were deposited by spin coating from 20 mg mL^–1^ solutions in chlorobenzene at various spin speeds to give around 100–150 nm thick active layers. MoO_3_ (10 nm) and Ag (100 nm) were evaporated through a shadow mask of active area 0.045 cm^2^ under base vacuum of 1 × 10^–6^ mbar. *J*–*V* characteristics were obtained using a xenon lamp with AM1.5G filters and 100 mW cm^–2^ illuminations (Oriel Instruments). EQE spectra were measured using a 100 W Tungsten–Halogen lamp. Simulated light was calibrated with a silicon photodiode as a reference; all the device measurements were taken behind a quartz window in a N_2_ filled container. The results are averaged from 12 devices. The PCE of the devices has not been confirmed from independent certification laboratories.

### Photoluminescence and electroluminescence spectra

PL measurements were performed by using a steady state spectrofluorometer (Horiba Jobin Yvon, Spex Fluoromax 1) exciting the samples at 445 nm. EL measurements were performed by using a Oriel SiCCD camera and a constant current density supplied by an external current/voltage source through the devices that have an active area of 0.104 cm^2^. IDTBR EL spectra was taken using an IHR2 InGaAs detector. The system was wavelength calibrated.

### FTPS

Devices are fabricated as outlined in the device fabrication part. FTPS-EQE measurements were carried out using a Vertex 70 from Bruker optics, equipped with QTH lamp, quartz beam splitter and external detector option. A low noise current amplifier (DLPCA-200) was used to amplify the photocurrent produced upon illumination of the photovoltaic devices with light modulated by the FTIR. The output voltage of the current amplifier was fed back to the external detector port of the FTIR, in order to use the FTIR's software to collect the photocurrent spectrum.

### Transient photovoltage

The experimental details are as previously described in ref. 40.

### Transient absorption spectroscopy

Microsecond and femtosecond TAS studies were carried out by using a Solstice (Newport Corporation) Ti:Sapphire regenerative amplifier (1 kHz repetition rate, 800 nm laser pulse with 92 fs pulse width). A part of the laser pulse was used to generate the pump pulse at 680 nm 4 μJ cm^–2^ with a TOPAS-Prime (Light conversion) optical parametric amplifier. TOPAS (Light Conversion Ltd) was used to generate the probe light in a visible continuum (450–800 nm) by a sapphire crystal. The spectra and decays were obtained by a HELIOS transient absorption spectrometer (800–1600 nm) and 6 ns resolution. The films were measured in N_2_ atmosphere to prevent degradation. Global analyses of the data were carried out using Origin and Matlab.

### Grazing incidence wide angle X-ray scattering measurements (GIWAXS)

GIWAXS experiments were performed at beam-line D1 at the Cornell High Energy Synchrotron Source, Wilson Lab, NY, USA. A fast 2D detector (PILATUS 200k from Dectris) has been used to record the scattering intensity with an exposure time of 1 second. An incident X-ray beam with a wavelength of 1.1555 Å and incident angle of 0.17° with respect to the sample plane was used to perform the experiments. The sample-to-detector-distance was 173.756 mm. Silver behenate (low angle diffraction standard) was used to calibrate the lengths in the reciprocal space.

### Simulations

Numerical simulations were carried out with MATLAB to estimate potential device efficiencies *η* = *J*_sc_ *V*_oc_FF/*P*_sun_. The short circuit current density is given by 
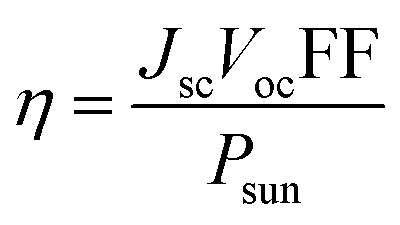
 where *φ*_sun_ denotes the photon number density of the solar AM1.5 spectrum. The EQE is set to the wavelength-independent value of EQE_max_ for energies larger than the band gap and to zero otherwise. The open-circuit voltage is expressed in terms of the voltage loss Δ*V*_oc_ = *E*_g_/*q* – *V*_oc_ which per definition yields *V*_oc_ = *E*_g_/*q* – Δ*V*_oc_. The upper limit for the fill factor FF of a solar cell is calculated from an ideal device without contact resistance and with an ideality factor of one (*n*_id_ = 1) for which the one-diode model and the superposition of dark and photocurrent *J*_ph_ is valid for all voltages. The current is then given by *J* = *J*_0_(e^*qV*(*kT*)^–1^^ – 1) – *J*_sc_ which allows the numerical maximization of the electrical output power *P*_el_ = –*J* × *V* yielding the maximum power point MPP. Under the given assumptions, the fill factor defined by 
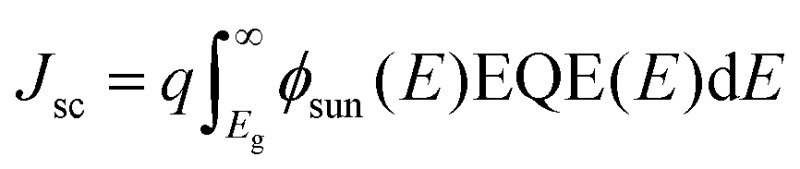
 only depends on one parameter – namely the *V*_oc_. Here, we use the approximate analytical expression FF = *v*_oc_ – ln(*v*_oc_ + 0.72)/(*v*_oc_ + 1) where *v*_oc_ = *qV*_oc_/(*kT*) which shows good accuracy for devices with reasonably high fill factors that are of interest here. In organic solar cells the FF is often significantly lower so that the FF itself is an important criterion for device optimization.^50^ Putting everything together the efficiency potential is – under the discussed assumptions regarding EQE and FF – defined by only three parameters: EQE_max_, Δ*V*_oc_ and *E*_g_. To show the efficiency potential as a function of EQE_max_ and Δ*V*_oc_ the band gap is optimized for each point in Fig. 6A. Fig. 6B shows linecuts of Fig. 6A along the indicated lines also shown in Fig. 1 which fix the relation between EQE_max_ and Δ*V*_oc_ – again using individually optimized band gaps. The grey line represents the empirical limit suggested by Li *et al.* ref. 4. The blue and violet lines result from the new data points presented in this work for FBR (EQE_max_ = 0.57, Δ*V*_oc_ = 0.5 V) and IDTBR (EQE_max_ = 0.75, Δ*V*_oc_ = 0.56 V) acceptors, respectively. The lines are chosen to meet the grey line in one point where Δ*V*_oc_ = 1 V and EQE_max_ = 0.9. Fig. 6C and D are based on the same linear relations between EQE_max_ and Δ*V*_oc_ given by the violet and blue line, respectively, which leaves *E*_g_ and Δ*V*_oc_ as the free parameters on the axis.

## Funding

S. H., A. W. and I. M. thanks EC FP7 Project SC2 (610115), EC FP7 Project ArtESun (604397), and EPSRC Project EP/G037515/1, EC FP7 Project POLYMED (612538). TK acknowledges support from the DFG (Grant KI-1571/2-1).

## Author contributions

S. H. synthesized the FBR acceptor. H. Yan supplied the polymer PffBT4T-2DT. D. B. fabricated and characterized solar cell devices and did the material characterizations. D. B. and N. G. carried out EL and FTPS measurements. D. B. and S. D. carried out TAS experiments. S. W. carried out TPV and CE measurements. M. A. carried out GIWAXS measurements. T. K. and P. K. made the calculations of the efficiency potential. J. G. prepared helped for literature data collection. D. B. and T. K. prepared the manuscript. All authors discussed the results and commented on the manuscript. A. A. supervised GIWAXS and J. R. D. supervised TAS, TPV and CE measurements. A. A., J. R. D., C. J. B. and I. M. revised the manuscript.

## Competing interests

There are no competing interests.
