# Supplementary material for: Reduced voltage losses yield 10% efficient fullerene free organic solar cells with >1 V open circuit voltages
Source: Energy Environ Sci. 2016 Nov 9;9(12):3783–93. doi: 10.1039/c6ee02598f (PMC5171224; doi:10.1039/c6ee02598f)
Supplement: Supplementary file 1 [file EE-009-C6EE02598F-s001.pdf]

Electronic Supplementary Material (ESI) for Energy & Environmental Science.
This journal is © The Royal Society of Chemistry 2016

Supplementary Information

Reduced voltage losses yield 10% and >1V fullerene free organic solar cells

Derya Baran*, Thomas Kirchartz*, Scot Wheeler, Stoichko Dimitrov, Maged Abdelsamie, Jeffrey Gorman, Raja Shahid Ashraf, Sarah Holliday, Andrew Wadsworth, Nicola Gasparini, Pascal Kaienburg, He Yan, Aram Amassian, Christoph J. Brabec, James R. Durrant, Iain McCulloch

Table S1. The correlation between E_g-qV_{oc} and EQE_{max} is shown from literature using polymers with different types of acceptors.

Ordered by increasing E_g-qV_{oc} . It is clear that when E_g-qV_{oc} < 0.6 V, there is a compromise between the EQE and the V_{oc}. Among all the donor:acceptor combinations PffBT4T-2DT:FBR is the only one which has V_{oc} > 1.0 V with *EQE*s >50% and E_g-qV_{oc} ≈ 0.5 V. PffBT4T-2DT:FBR is shown in red. The combinations with V_{oc} higher than 1 V are shown in green.

Donor: Small Molecule

**Donor**	**Acceptor**	**Donor E_g (eV)**	**V_{oc} (mV)**	**E_g-qV_{oc} (eV)**	**EQE_{max}**	**Supplementary Ref.**
P*ff*BT4T-2DT	FBR	1.6	1090	0.48-0.5	0.57	(*1*)
PTB7-Th	SF-PDI_2	1.58	1000	0.58	0.35	(*2*)
p-DTS$(FBTTh)_2$	S1	1.5	910	0.59	0.15	(*3*)
PTB7-Th	IEIC	1.58	967	0.613	0.57	(*2*)
PBDTTT-C-T	DC-IDT2F	1.58	960	0.62	0.33	(*4*)
p-DTS$(FBTTh)_2$	S4	1.5	870	0.63	0.18	(*3*)
PBDTTT-C-T	DC-IDT2F	1.58	940	0.64	0.28	(*4*)
DPP-Py	BT$(TPI\text{-}EH)_2$	1.72	1070	0.65	0.12	(*5*)
p-DTS$(FBTTh)_2$	NIDCS-MO	1.5	850	0.65	0.47	(*6*)
p-DTS$(FBTTh)_2$	S3	1.5	840	0.66	0.05	(*3*)
DPP-Py	BT$(TTI\text{-}n12)_2$	1.72	1050	0.67	0.28	(*5*)
PTB7-Th	TPE-PDI_4	1.58	910	0.67	0.56	(*7*)
P*ff*BT4T-2DT	SF-PDI_2	1.65	980	0.67	0.5	(*2*)
P3HT	DIR-2EH	1.9	1220	0.68	0.35	(*8*)
PBDTTT-C-T	DC-IDT2T	1.58	900	0.68	0.4	(*9*)
P*ff*BT4T-2DT	TPC-PDI_4	1.65	960	0.69	0.46	(*10*)
P3HT	PFTBT	1.9	1200	0.7	0.34	(*11*)
DPP-Py	BT$(TPI\text{-}n12)_2$	1.72	1010	0.71	0.14	(*5*)
PBDTTT-C-T	S(TPA-PDI)	1.58	870	0.71	0.51	(*12*)

P*ff*BT4T-2DT	TPSi-PDI_4	1.65	940	0.71	0.44	(*10*)
P3HT	$F(DPP)_2B_2$	1.9	1180	0.72	0.25	(*13*)
SubPc	EBB	2.1	1380	0.72	0.36	(*14*)
PPDT2FBT	NIDCS-HO	1.76	1030	0.73	0.7	(*15*)
P*ff*BT4T-2DT	TPGe-PDI_4	1.65	920	0.73	0.31	(*10*)
PBDTTT-C-T	IDTT-2BM	1.58	845	0.735	0.5	(*16*)
p-DTS$(FBTTh)_2$	PDI-2DTT	1.5	763	0.737	0.46	(*17*)
PBDTTT-C-T	*bis*-PDI-T-EG	1.58	840	0.74	0.59	(*18*)
PBDTTT-C-T	Compound-3	1.58	840	0.74	0.11	(*19*)
P3HT	P2	1.9	1150	0.75	0.04	(*20*)
P3HT	$SF(DPPB)_4$	1.9	1140	0.76	0.48	(*21*)
P3HT	IDT-2DDP	1.9	1130	0.77	0.11	(*22*)
P3HT	$PhDMe(DPP)_2$	1.9	1130	0.77	0.13	(*23*)
DPP-Py	$BT(TTI\text{-}EH)_2$	1.72	950	0.77	0.2	(*5*)
PTB7-Th	ITIC	1.58	810	0.77	0.74	(*24*)
PTB7-Th	di-PDI	1.58	800	0.78	0.67	(*25*)
PTB7-Th	hPDI3	1.58	800	0.78	0.7	(*18*)
PTB7-Th	hPDI4	1.58	800	0.78	0.78	(*18*)
PTB7-Th	Helical-PDI	1.58	796	0.784	0.58	(*26*)
PBTI3T	Phenyl-PDI	1.81	1024	0.786	0.46	(*27*)
PBDTTT-C-T	bis-PDI-Se-EG	1.58	790	0.79	0.48	(*28*)
P3HT	DPP1	1.9	1100	0.8	0.17	(*29*)
P3HT	Ph(DPP)2	1.9	1100	0.8	0.09	(*23*)
DPP-Py	ITNCN(TPI-n12)2	1.72	920	0.8	0.14	(*5*)
PBDTTT-C-T	bis-PDI-T-MO	1.58	780	0.8	0.45	(*30*)
PBDTTT-C-T	TPDI	1.58	770	0.81	0.47	(*31*)
PBDTTT-C-T	Me-PDI	1.58	770	0.81	0.35	(*32*)
P*ff*BT4T-2DT	di-PDI	1.65	840	0.81	0.55	(*2*)
PBDTTT-C-T	PDI-2DTT	1.58	761	0.819	0.39	(*17*)
P3HT	N6	1.9	1080	0.82	0.016	(*33*)
p-DTS$(FBTTh)_2$	S2	1.5	680	0.82	0.09	(*3*)
PBDTTT-C-T	IDT-2BM	1.58	760	0.82	0.51	(*16*)
P3HT	9, 9' BF	1.9	1070	0.83	0.302	(*34*)
PTB7	DTBT(TDPP)2	1.65	820	0.83	0.42	(*35*)
DPP-Py	ITNCN(TPI-EH)2	1.72	890	0.83	0.21	(*5*)
PTB7	DTDfBT(TDPP)2	1.65	810	0.84	0.68	(*35*)
P3HT	Flu-RH	1.9	1030	0.87	0.41	(*36*)
P3HT	Cz-RH	1.9	1030	0.87	0.38	(*36*)
PTB7	hPDI4	1.65	780	0.87	0.7	(*18*)
P3HT	PMI-F	1.9	1020	0.88	0.21	(*37*)
P3HT	PMI-F	1.9	1020	0.88	0.21	(*37*)

PTB7	hPDI3	1.65	760	0.89	0.71	(*18*)
PSEHTT	DBFI-MTT	1.82	930	0.89	0.48	(*38*)
P*ff*T2-FTAZ-2DT	IEIC	1.907692	998	0.909692	0.57	(*39*)
PSEHTT	DBFI-DMT	1.82	910	0.91	0.8	(*38*)
P3HT	CSORG5	1.9	980	0.92	0.55	(*40*)
P3HT	PMIF-PMI	1.9	970	0.93	0.3	(*37*)
P3HT	DBS-2DPP	1.9	970	0.93	0.3	(*41*)
P3HT	PMI-F-PMI	1.9	970	0.93	0.3	(*37*)
P3HT	HPI-BT	1.9	960	0.94	0.41	(*42*)
P3HT	Ph-CN	1.9	960	0.94	0.41	(*43*)
P3HT	*p*-CH3Ph-CN	1.9	960	0.94	0.32	(*43*)
P3HT	P1	1.9	960	0.94	0.13	(*20*)
P3HT	*o*-CH3Ph-CN	1.9	950	0.95	0.47	(*43*)
P3HT	FEHIDT	1.9	950	0.95	0.17	(*44*)
PDBT-T1	Sdi-PBI-S	1.85	900	0.95	0.7	(*45*)
P3HT	α-Naph-CN	1.9	940	0.96	0.41	(*43*)
P3HT	*o*-DPP-PhCN	1.9	940	0.96	0.125	(*46*)
PSEHTT	DBFI-T	1.82	860	0.96	0.65	(*47*)
P3HT	β-Naph-CN	1.9	930	0.97	0.34	(*43*)
SF8TBT	TTzBT-DCAO	2.21	1240	0.97	0.48	(*48*)
PSEHTT	DBFI-S	1.82	820	1	0.24	(*38*)
P3HT	Th-Cn	1.9	880	1.02	0.34	(*43*)
P3HT	IDT-2BR	1.9	840	1.06	0.47	(*49*)
P3HT	Cor-PI	1.9	830	1.07	0.24	(*50*)
P3HT	FBR	1.9	820	1.08	0.79	(*51*)
P3HT	Cor-NI	1.9	820	1.08	0.075	(*50*)
P3HT	m-DPP-PhCN	1.9	730	1.17	0.05	(*46*)
P3HT	F8IDT	1.9	720	1.18	0.3	(*44*)
P3HT	IDT-PDI	1.9	700	1.2	0.36	(*52*)
P3HT	BDP-BDT	1.9	650	1.25	0.13	(*53*)
P3HT	B3	1.9	640	1.26	0.27	(*54*)
P3HT	P(NDI-TCPDTT)	1.9	630	1.27	0.02	(*55*)
P3HT	BDP-CPDT	1.9	620	1.28	0.1	(*53*)
P3HT	K12	1.9	620	1.28	0.27	(*56*)
P3HT	*bis*-PDI-Se-EG	1.9	590	1.31	0.28	(*28*)
P3HT	BDP-DTP	1.9	570	1.33	0.1	(*53*)
P3HT	MHPI-BTCN	1.9	570	1.33	0.07	(*57*)
P3HT	*p*-DPP-PhCN	1.9	560	1.34	0.13	(*46*)
P3HT	P(NDIOD-T2)	1.9	560	1.34	0.05	(*55*)
P3HT	F4PI-BTCN	1.9	530	1.37	0.2	(*57*)
P3HT	F4PI-$(BTCN)_2$	1.9	470	1.43	0.19	(*57*)

PInCz	HPI-BT	2.8	1350	1.45	0.16	(*58*)
P3HT	PhPI-BTCN	1.9	230	1.67	0.16	(*57*)

Donor:Fullerene

**Donor**	**Acceptor**	**Donor E_g (eV)**	**V_{oc} (mV)**	**E_g-qV_{oc} (eV)**	**EQE_{max}**	**Supplementary Ref.**
P*ff*BT4T-2OD	PC71BM	1.44	960	0.48	0.11	(*59*)
PNT4T-2OD	PC71BM	1.47	920	0.55	0.4	(*59*)
PTB7	PC71BM	1.53	980	0.55	0.25	(*59*)
PBDTTT-EFF	PC71BM	1.22	660	0.56	0.65	(*60*)
PTB7-Th	PC71BM	1.52	960	0.56	0.64	(*61*)
PBDTTT-EFS	PC71BM	1.28	690	0.59	0.52	(*59*)
P3HT	PC61BM	1.5	910	0.59	0.32	(*62*)
P3HT	ICMA	1.34	720	0.62	0.42	(*63*)
P3HT	ICBA	1.4	770	0.63	0.56	(*64*)
PDBT-T1	PC71BM	1.5	860	0.64	0.6	(*65*)
P3HT	*bis*-PC61BM	1.45	800	0.65	0.21	(*65*)
P3	PC71BM	1.36	710	0.65	0.24	(*63*)
P4	PC61BM	1.38	720	0.66	0.46	(*66*)
LBPP-6	PC61BM	1.38	720	0.66	0.5	(*66*)
LBPP-6	PC71BM	1.51	850	0.66	0.51	(*67*)
PFPDT	PC61BM	1.46	780	0.68	0.47	(*64*)
PFPDT	PC71BM	1.43	750	0.68	0.62	(*68*)
PBDTTT-CF	PC71BM	1.35	670	0.68	0.79	(*69*)
PSBTBT	PC71BM	1.36	680	0.68	0.4	(*63*)
PDTPBT-C8	PC61BM	1.6	920	0.68	0.28	(*70*)
DT-PDPP2T-TT	PC71BM	1.6	920	0.68	0.74	(*71*)
PDPPFTF	PC71BM	1.38	698	0.682	0.75	(*72*)
PDPP2TzT	PC71BM	1.39	708	0.682	0.48	(*73*)
PDPP2TzBDT	PC71BM	1.38	690	0.69	0.49	(*64*)
PDPP2Tz2T	PC71BM	1.37	680	0.69	0.36	(*63*)
PDPP2TzDTP	PC71BM	1.6	910	0.69	0.45	(*74*)
TQ1	PC71BM	1.38	680	0.7	0.62	(*67*)
PCPDTBT	PC71BM	1.61	910	0.7	0.7	(*75*)
PDTG-TPD	PC71BM	1.3	600	0.7	0.61	(*76*)
P3TI	PC71BM	1.33	630	0.7	0.45	(*77*)
PThTPTI	PC71BM	1.3	600	0.7	0.47	(*77*)
PiITVT	PC61BM	1.28	580	0.7	0.6	(*77*)
PPDTBT	PC71BM	1.51	800	0.71	0.71	(*78*)
PPDTFBT	PC71BM	1.37	660	0.71	0.45	(*63*)
PPDT2FBT	PC71BM	1.6	890	0.71	0.28	(*79*)
PBDTTPD(2EH/C8)	PC71BM	1.45	730	0.72	0.41	(*80*)

PBDTT-TT	PC71BM	1.57	840	0.73	0.67	(*81*)
PBDTT-O-TT	PC71BM	1.69	950	0.74	0.69	(*82*)
PBDTT-S-TT	PC71BM	1.49	750	0.74	0.46	(*66*)
PCPDTBT	PC71BM	1.52	780	0.74	0.68	(*83*)
PCPDT-DFBT	PC71BM	1.62	880	0.74	0.69	(*84*)
PPDT-DFBT	PC71BM	1.45	700	0.75	0.42	(*85*)
PMDPP3T	PC71BM	1.4	650	0.75	0.36	(*86*)
PIDTT-DFBT-T	PC71BM	1.4	650	0.75	0.41	(*86*)
PIDTT-DFBT-TT	PC71BM	1.4	650	0.75	0.48	(*86*)
PDTSTPD	PC71BM	1.13	380	0.75	0.39	(*87*)
PBDT-T8-TPD	PC61BM	1.61	860	0.75	0.58	(*71*)
PDPP5T	PC61BM	1.45	690	0.76	0.33	(*80*)
PDPP4TP	PC71BM	1.42	660	0.76	0.65	(*88*)
PDPP4TOP	PC71BM	1.57	810	0.76	0.3	(*88*)
C1	PC71BM	1.53	770	0.76	0.51	(*88*)
C3	PC71BM	1.53	760	0.77	0.79	(*89*)
PDPP4TN	PC71BM	1.76	990	0.77	0.67	(*90*)
PDPP4TP	PC71BM	1.58	807	0.773	0.66	(*91*)
PDPP4TBDT	PC71BM	1.43	650	0.78	0.5	(*88*)
PDPP4TTT	PC71BM	1.76	980	0.78	0.65	(*90*)
PDPP6T	PC71BM	1.7	910	0.79	0.68	(*92*)
PDPP5T	PC71BM	1.43	640	0.79	0.6	(*88*)
PBDPP-1	PC71BM	1.75	960	0.79	0.69	(*93*)
PBDPP-2	PC71BM	1.58	784	0.796	0.71	(*91*)
PBDPP-3	PC71BM	1.5	700	0.8	0.77	(*94*)
PTPD3T	PC71BM	1.43	630	0.8	0.6	(*80*)
PBTIT3T	PC71BM	1.5	700	0.8	0.66	(*94*)
DT-PDPPTPT	PC71BM	1.53	730	0.8	0.7	(*81*)
DT-PDPP3T	PC71BM	1.37	570	0.8	0.61	(*95*)
DT-PDPP2T-DBT	PC71BM	1.43	620	0.81	0.52	(*96*)
DT-PDPP2T-BDT	PC71BM	1.58	770	0.81	0.65	(*81*)
DT-PDPP4T	PC71BM	1.63	810	0.82	0.48	(*64*)
DT-PDPP2T-TT	PC71BM	1.51	690	0.82	0.65	(*97*)
PIPCP	PC61BM	1.8	980	0.82	0.79	(*98*)
PIPC-RA	PC61BM	1.74	910	0.83	0.79	(*99*)
PIT	PC71BM	1.7	860	0.84	0.74	(*100*)
PR1	PC71BM	1.46	610	0.85	0.7	(*101*)
PR2	PC71BM	1.85	1000	0.85	0.56	(*102*)
PR3	PC71BM	1.56	708	0.852	0.78	(*103*)
PBDTFBZS	PC71BM	1.68	820	0.86	0.61	(*104*)
PBT-TBDTT	PC71BM	1.48	620	0.86	0.45	(*67*)

PBTT-TBDTT	PC71BM	1.83	970	0.86	0.71	(*105*)
PDPP3T-*alt*-TPT	PC71BM	1.46	590	0.87	0.57	(*101*)
PBDTT-FDPP-C12	PC61BM	1.78	910	0.87	0.54	(*93*)
PBDTT-SeDPP	PC61BM	1.54	670	0.87	0.61	(*106*)
PDTP-DFBT	PC71BM	1.47619	600	0.88	0.54	(*101*)
BTT-DPP	PC71BM	1.65	770	0.88	0.82	(*89*)
P1	PC71BM	1.46	580	0.88	0.64	(*107*)
P2	PC71BM	1.25	370	0.88	0.48	(*95*)
P3	PC71BM	1.6	720	0.88	0.77	(*108*)
P4	PC71BM	1.66	760	0.9	0.44	(*88*)
PDTTDPP	PC71BM	1.76	860	0.9	0.82	(*109*)
PDPP2FT-C12	PC71BM	1.65	740	0.91	0.666	(*110*)
PDPP2FT-C14	PC71BM	1.72	810	0.91	0.69	(*109*)
PDPP2FT-C16	PC71BM	1.72	810	0.91	0.7	(*109*)
PTTDPO	PC71BM	1.84	930	0.91	0.74	(*111*)
PTTDPS	PC71BM	1.83	910	0.92	0.73	(*112*)
P1	PC71BM	1.81	880	0.93	0.55	(*113*)
P2	PC71BM	1.81	880	0.93	0.76	(*112*)
PTI-1	PC61BM	1.82	890	0.93	0.76	(*114*)
PTI-1	PC71BM	1.45	510	0.94	0.69	(*106*)
P3TI	PC71BM	1.82	850	0.97	0.76	(*115*)
PM6	PC71BM	1.9	920	0.98	0.44	(*116*)
PNNT (12HD)	PC71BM	1.86	870	0.99	0.76	(*117*)
TBTIT-h	PC71BM	1.82	813	1.007	0.35	(*118*)
PBDT-TS1	PC71BM	1.41	400	1.01	0.18	(*119*)
PTP-1	PC71BM	1.64	630	1.01	0.62	(*120*)
PDPT-DFBT	PC71BM	1.81	793	1.017	0.34	(*118*)
PDTP-DTDPP	PC71BM	1.7	680	1.02	0.59	(*85*)
PNTz4T	PC71BM	1.82	786	1.034	0.76	(*115*)
PNOz4T	PC71BM	1.9	840	1.06	0.61	(*121*)
DRCN8T	PC71BM	1.74	670	1.07	0.52	(*120*)
DRCN8TT	PC71BM	1.74	670	1.07	0.63	(*120*)
DBP	C60	1.65	560	1.09	0.65	(*120*)
CuPc	C60	1.66	560	1.1	0.56	(*120*)
DPSQ	C60	1.6	500	1.1	0.76	(*122*)
o-BDTdFBT	PC71BM	1.71	560	1.15	0.59	(*120*)
BTR	PC71BM	1.9	685	1.215	0.55	(*123*)
DR3TSBDT	PC71BM	1.9	630	1.27	0.61	(*121*)
DCV5T-Me(3)	C60	1.9	580	1.32	0.65	(*121*)
DRCN5T	PC71BM	2	520	1.48	0.34	(*124*)
DTS(PTTh$_2$)$_2$	PC71BM	2	480	1.52	0.24	(*124*)

Donor:Polymer

Donor	Acceptor	Donor E_g (eV)	V_{oc} (mV)	E_g-qV_{oc} (eV)	EQE_{max}	Supplementary Ref.
PDPP2Tz-T	PDPP5Y	1.44	800	0.64	0.21	(*125*)
PDPP5T	PDPP2TzT	1.45	810	0.64	0.29	(*125*)
POPT	CN-PPV	1.8	1040	0.76	0.22	(*126*)
PTB7-Th	P(PDI-BDT-T)	1.58	800	0.78	0.55	(*127*)
NT	N2200	1.58	770	0.81	0.52	(*128*)
PSBTBT	PDI-DTT	1.45	596	0.854	0.082	(*129*)
M3EH-PPV	CN-ether-PPV	2.4	1360	1.04	0.31	(*130*)
PSEHTT	PNDIS-HD	1.82	760	1.06	0.47	(*131*)
PSEHTT	PNDIS	1.82	750	1.07	0.38	(*131*)
PT1	PC-PDI	1.82	740	1.08	0.43	(*132*)
P3HT	PC-NDI	1.9	700	1.2	0.1	(*133*)
PSEHTT	PNDIT	1.82	610	1.21	0.28	(*131*)
P3HT	PF-NDI	1.9	680	1.22	0.3	(*134*)

Table S2: Film absorption maxima, ionization potential (IP), electron affinity (EA), band gaps determined from cyclic voltammetry ($E_{g,CV}$) from the onsets of oxidation and reduction potential measured, optical band gaps $E_{g,opt}$, calculated from the absorption edges for PffBT4T-2DT, FBR, FTTB, EH-IDFBR, PCBM,IDTBR and PffBT4T-2DT:FBR. * Estimated from optical band gaps.

	λmax film (nm)	IP (eV) (±0.1)	EA (eV) (±0.1)	$E_{g,CV}$ (eV) (±0.1)	$E_{g,opt}$ (eV) (±0.01)	τ (ps)
PffBT4T-2DT	694	5.34	3.70*	-	1.61	237±12
FBR	510	5.83*	3.75	-	2.08	-
EH-IDFBR	505	5.75*	3.70		2.15	
FTTB	545	5.65*	3.62		2.04	
IDTBR	690	5.5	3.88		1.6	
PCBM	333	6.1*	4.0		1.70	-
PffBT4T-2DT :FBR	485	5.32	3.74	1.58	1.60	48±7

Table S3: Photovoltaic performances of the solar cells based on PffBT4T-2DT:NFA under standard AM 1.5G illumination. * surface of ZnO is modified with washing the layer with the solvent of zinc acetate. [a] Short circuit density measured from *J-V* measurements. [b] PCE_{ave}: average power conversion efficiency with standard deviation from 12 devices.

PffBT4T-2DT:FBR	J_{sc} [a](mA/cm^2)	V_{oc} (V)	*FF* (%)	PCE_{ave} [b](%)	*EQE* @λ_{max} (%)
1:0.8	8.1	1.0	59	4.8 (±0.20)	35
1:1	11.6	1.09	60	7.7 (±0.18)	57
1:1*	11.5	1.12	61	7.8 (± 0.20)	57
1:1.5	10.2	1.10	56	6.3 (± 0.20)	45
1:2	10.3	1.06	54	5.9 (±0.18)	45
PffBT4T-2DT: PCBM					
1:2 (3% DIO)	16	0.76	62	7.5 (±0.3)	70
PffBT4T-2DT: FTTB (1:1)	10.3	1.05	45	5.0 (±0.2)	-
PffBT4T-2DT: EH-IDFBR (1:1)	4.4	1.08	38	1.75 (±0.2)	-
PffBT4T-2DT: IDTBR (1:1)	15	1.07	62	9.95 (±0.2)	76

Table S4. PffBT4T-2DT singlet exciton lifetimes in neat polymer film and PffBT4T-2DT:FBR blend and the calculated and yield of charge generation for the blend. The exciton lifetime was extracted from fitting the transient absorption kinetics with exponential and stretched exponential functions. The latter is commonly used to estimate the rate of charge transfer in dispersive donor-acceptor systems with large acceptor density of states. The error bars represent the standard deviation from the best fit. The P3HT exciton lifetime and generation yield of P3HT:FBR blend is included in the table as a reference. P3HT data is taken from ref [53].

	**Lifetime [ps]**	**Yield charge generation [%]**
PffBT4T-2DT	237 ± 12	~
PffBT4T-2DT:FBR	48 ± 7	80 ± 4
P3HT:FBR	<1	96

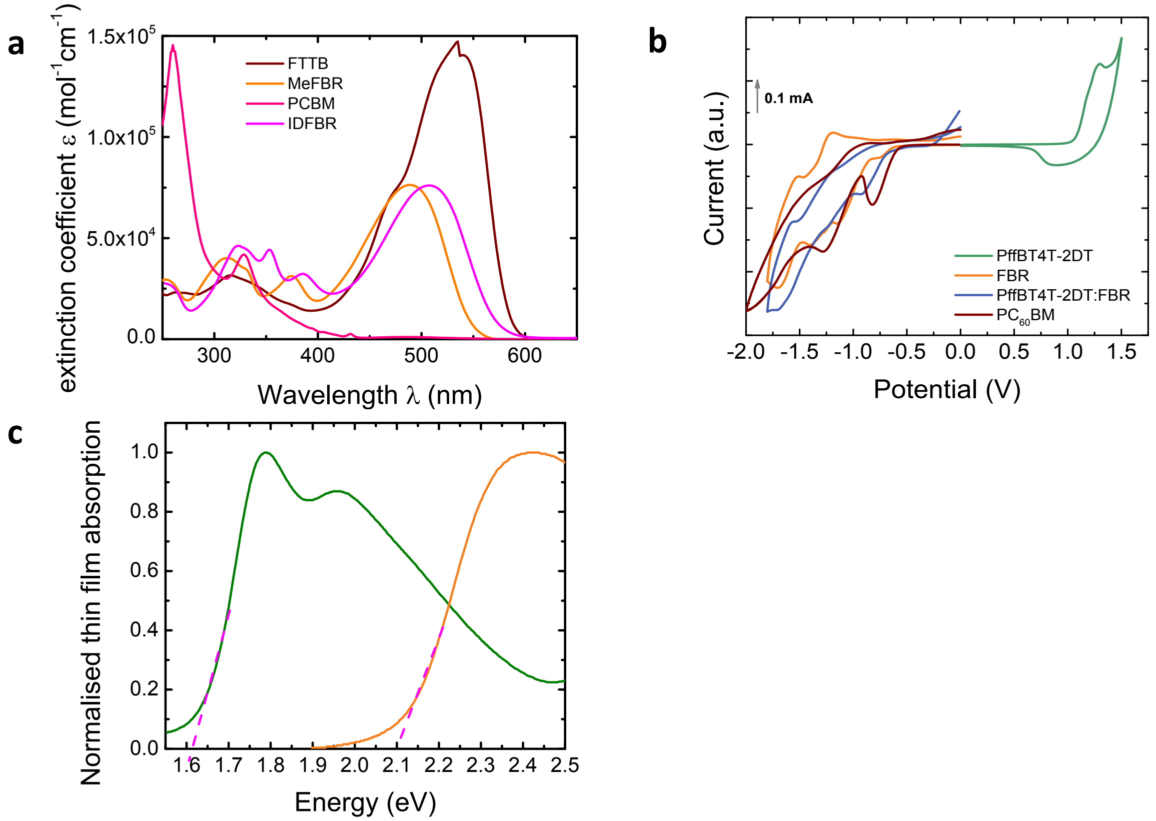

Figure S 1 **a** Absorption coefficient data for PffBT4t-2DT and FBR films, **b** Cyclic voltammograms of neat PffBT4T-2DT, FBR and PC60BM films and PffBT4T-2DT:FBR blend film in 0.1 M TBAPF-6 /acetonitrile system with 50 mV/s scan rate using Ag/AgCl reference electrode, **c** Absorption edges of FBR and PffBT4T-2DT for optical band gap calculation.

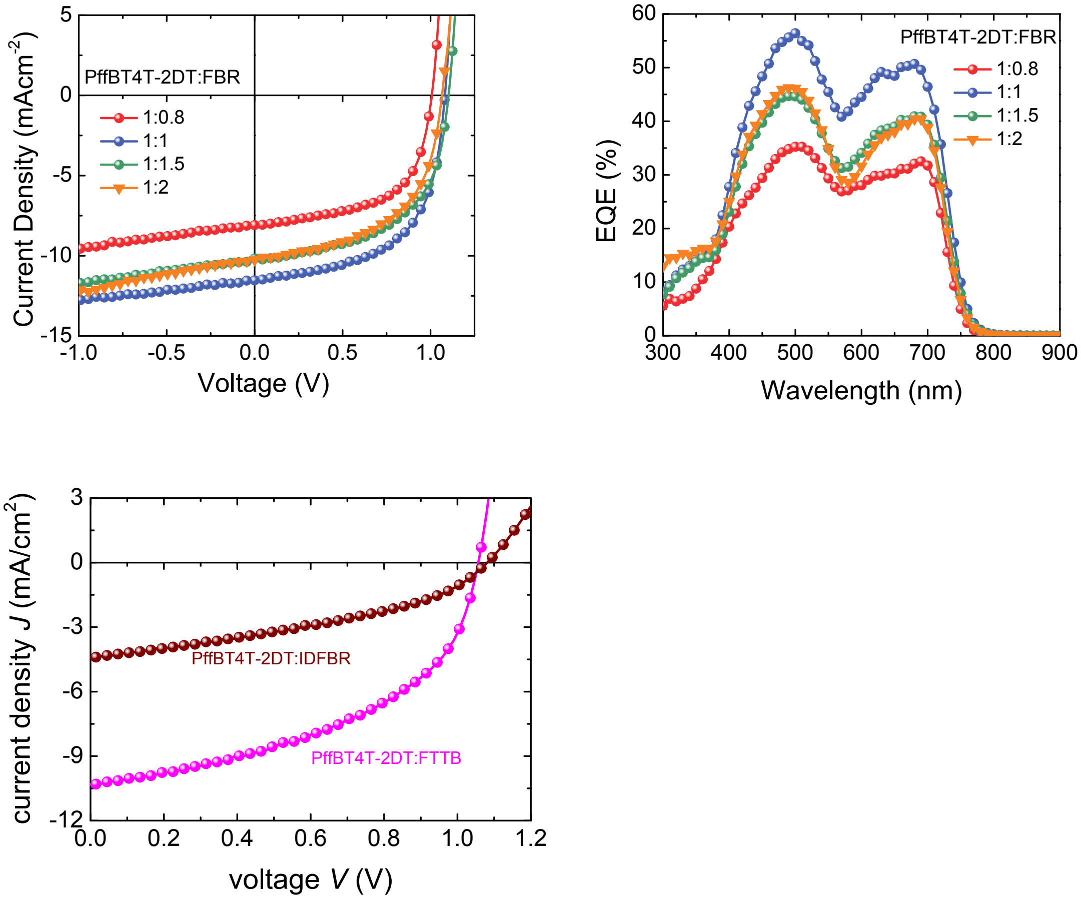

Figure S 2 **a** Current density - voltage curves of PffBT4T-2DT:FBR devices with different donor:acceptor ratios under illumination of $100mWcm^{-2}$. **b** *EQE* spectra of corresponding PffBT4T-2DT:FBR devices and **c** Current density - voltage curves of PffBT4T-2DT:FTTB and PffBT4T-2DT:IDFBR devices under illumination of $100mWcm^{-2}$.

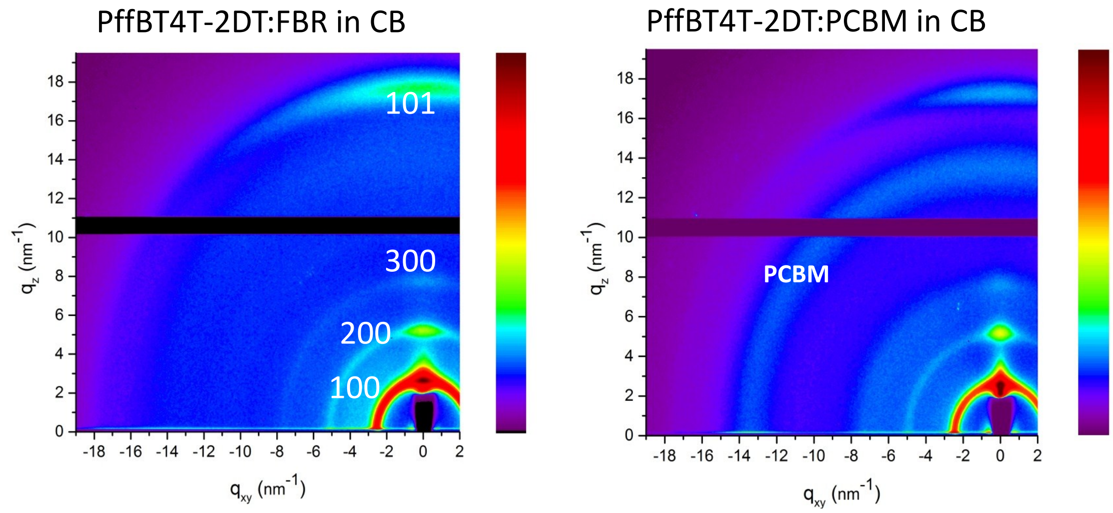

Figure S 3 2D GIWAX patterns of PffBT4T-2DT:FBR and PffBT4T-2DT:PCBM, respectively.

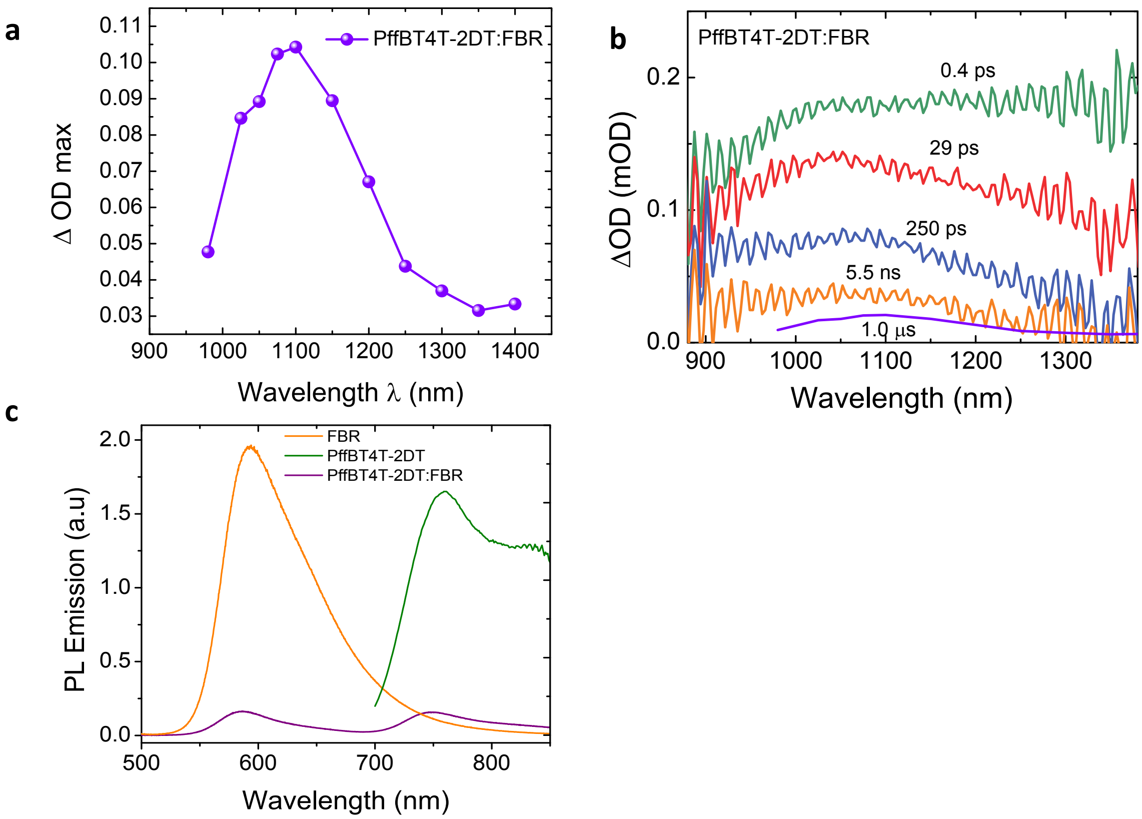

Figure S 4 **a** femto-second transient absorption spectra of PffBT4T-2DT:FBR recorded after 680 nm light excitation in the NIR spectral region from 400 fs to 1 µs. The spectra consist of two spectral signatures of the 1300 nm absorption peak corresponding to singlet polymer excitons and 1000 nm polaron peak and **b** micro-second TAS profile of PffBT4T-2DT:FBR excited at different wavelengths between 950 nm and 1400 nm. The polaron peak is observed around 1100 nm and **c** Photoluminescence (PL) spectra based on neat PffBT4T-2DT, FBR and PffBT4T-2DT:FBR blend (1:1 w/w) films. The films were excited with 445 nm for FBR and PffBT4T-2DT:FBR, and 680 nm for PffBT4T-2DT. Each spectrum was corrected for the absorption of the film at the excitation wavelength. The emission maxima of the blend quenched 86 ± 2 % relative to neat films which is consistent with the TAS measurements.

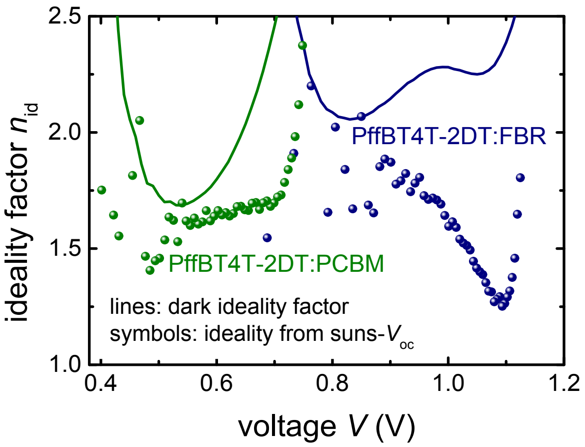

Figure S 5 Ideality factor values as a function of voltage for PffBT4T-2DT:FBR and PffBT4T-2DT:PCBM.

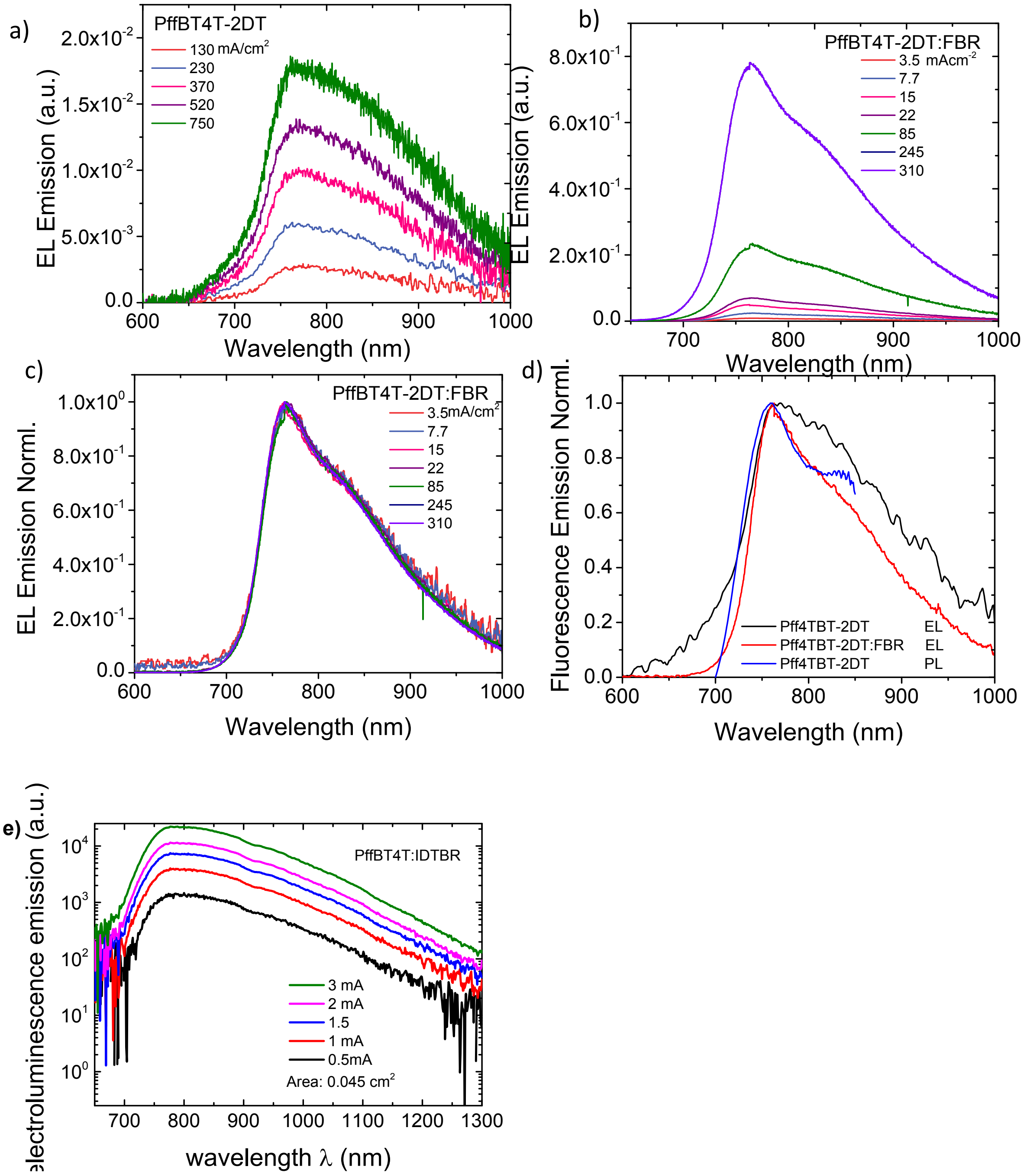

Figure S 6 Electroluminescence spectra of a) neat PffBT4T-2DT and b) PffBT4T-2DT:FBR devices at various injection currents. c) Normalized electroluminescence of PffBT4T-2DT:FBR devices exhibiting no shift upon higher injected current. d) Normalized emission data for neat and blend materials, showing EL from the blend is not different than PL and EL of neat PffBT4T-2DT. All spectra were measured using SiCCD.

References

1. This Work.
2. J. Zhao *et al.*, High-efficiency non-fullerene organic solar cells enabled by a difluorobenzothiadiazole-based donor polymer combined with a properly matched small molecule acceptor. *Energy & Environmental Science* **8**, 520-525 (2015).
3. S. M. McAfee, J. M. Topple, J.-P. Sun, I. G. Hill, G. C. Welch, The structural evolution of an isoindigo-based non-fullerene acceptor for use in organic photovoltaics. *RSC Advances* **5**, 80098-80109 (2015).
4. Y. Wang, H. Bai, X. Zhan, Comparison of conventional and inverted structures in fullerene-free organic solar cells. *Journal of Energy Chemistry* **24**, 744-749 (2015).
5. J. D. Douglas *et al.*, Solution-Processed, Molecular Photovoltaics that Exploit Hole Transfer from Non-Fullerene, n-Type Materials. *Advanced Materials* **26**, 4313-4319 (2014).
6. O. K. Kwon, J.-H. Park, D. W. Kim, S. K. Park, S. Y. Park, An All-Small-Molecule Organic Solar Cell with High Efficiency Nonfullerene Acceptor. *Advanced Materials* **27**, 1951-1956 (2015).
7. Y. Liu *et al.*, A Tetraphenylethylene Core-Based 3D Structure Small Molecular Acceptor Enabling Efficient Non-Fullerene Organic Solar Cells. *Advanced Materials* **27**, 1015-1020 (2015).
8. H.-Y. Chen *et al.*, Diindeno[1,2-g:1',2'-s]rubicene: all-carbon non-fullerene electron acceptor for efficient bulk-heterojunction organic solar cells with high open-circuit voltage. *RSC Advances* **5**, 3381-3385 (2015).
9. H. Bai *et al.*, An electron acceptor based on indacenodithiophene and 1,1-dicyanomethylene-3-indanone for fullerene-free organic solar cells. *Journal of Materials Chemistry A* **3**, 1910-1914 (2015).
10. Y. Liu *et al.*, Efficient non-fullerene polymer solar cells enabled by tetrahedron-shaped core based 3D-structure small-molecular electron acceptors. *Journal of Materials Chemistry A* **3**, 13632-13636 (2015).
11. C. Guo *et al.*, Conjugated Block Copolymer Photovoltaics with near 3% Efficiency through Microphase Separation. *Nano Letters* **13**, 2957-2963 (2013).
12. Y. Lin *et al.*, A Star-Shaped Perylene Diimide Electron Acceptor for High-Performance Organic Solar Cells. *Advanced Materials* **26**, 5137-5142 (2014).
13. H. Shi, W. Fu, M. Shi, J. Ling, H. Chen, A solution-processable bipolar diketopyrrolopyrrole molecule used as both electron donor and acceptor for efficient organic solar cells. *Journal of Materials Chemistry A* **3**, 1902-1905 (2015).
14. P. Sullivan *et al.*, An N-ethylated barbituric acid end-capped bithiophene as an electron-acceptor material in fullerene-free organic photovoltaics. *Chemical Communications* **51**, 6222-6225 (2015).
15. O. K. Kwon *et al.*, A High Efficiency Nonfullerene Organic Solar Cell with Optimized Crystalline Organizations. *Advanced Materials*, n/a-n/a (2015).
16. H. Bai *et al.*, Non fullerene acceptors based on extended fused rings flanked with benzothiadiazolylmethylenemalononitrile for polymer solar cells. *Journal of Materials Chemistry A* **3**, 20758-20766 (2015).
17. P. Cheng *et al.*, Towards high-efficiency non-fullerene organic solar cells: Matching small molecule/polymer donor/acceptor. *Organic Electronics* **15**, 2270-2276 (2014).
18. Y. Zhong *et al.*, Molecular helices as electron acceptors in high-performance bulk heterojunction solar cells. *Nat Commun* **6**, (2015).
19. S. Dai *et al.*, Perylene diimide–thienylenevinylene-based small molecule and polymer acceptors for solution-processed fullerene-free organic solar cells. *Dyes and Pigments* **114**, 283-289 (2015).

20. X. Liu *et al.*, Star-shaped isoindigo-based small molecules as potential non-fullerene acceptors in bulk heterojunction solar cells. *New Journal of Chemistry* **39**, 8771-8779 (2015).
21. S. Li *et al.*, A spirobifluorene and diketopyrrolopyrrole moieties based non-fullerene acceptor for efficient and thermally stable polymer solar cells with high open-circuit voltage. *Energy & Environmental Science*, (2015).
22. H. Bai *et al.*, A bipolar small molecule based on indacenodithiophene and diketopyrrolopyrrole for solution processed organic solar cells. *Journal of Materials Chemistry A* **2**, 778-784 (2014).
23. F. Zhang *et al.*, The effect of molecular geometry on the photovoltaic property of diketopyrrolopyrrole based non-fullerene acceptors. *Synthetic Metals* **203**, 249-254 (2015).
24. Y. Lin *et al.*, An Electron Acceptor Challenging Fullerenes for Efficient Polymer Solar Cells. *Advanced Materials* **27**, 1170-1174 (2015).
25. Y. Zang *et al.*, Integrated Molecular, Interfacial, and Device Engineering towards High-Performance Non-Fullerene Based Organic Solar Cells. *Advanced Materials* **26**, 5708-5714 (2014).
26. Y. Zhong *et al.*, Efficient Organic Solar Cells with Helical Perylene Diimide Electron Acceptors. *Journal of the American Chemical Society* **136**, 15215-15221 (2014).
27. P. E. Hartnett *et al.*, Slip-Stacked Perylenediimides as an Alternative Strategy for High Efficiency Nonfullerene Acceptors in Organic Photovoltaics. *Journal of the American Chemical Society* **136**, 16345-16356 (2014).
28. X. Zhang, J. Yao, C. Zhan, A selenophenyl bridged perylene diimide dimer as an efficient solution-processable small molecule acceptor. *Chemical Communications* **51**, 1058-1061 (2015).
29. H. Patil *et al.*, A non-fullerene electron acceptor based on fluorene and diketopyrrolopyrrole building blocks for solution-processable organic solar cells with an impressive open-circuit voltage. *Physical Chemistry Chemical Physics* **16**, 23837-23842 (2014).
30. Z. Lu *et al.*, Perylene–Diimide Based Non-Fullerene Solar Cells with 4.34% Efficiency through Engineering Surface Donor/Acceptor Compositions. *Chemistry of Materials* **26**, 2907-2914 (2014).
31. R. Shivanna *et al.*, Charge generation and transport in efficient organic bulk heterojunction solar cells with a perylene acceptor. *Energy & Environmental Science* **7**, 435-441 (2014).
32. W. Chen *et al.*, A perylene diimide (PDI)-based small molecule with tetrahedral configuration as a non-fullerene acceptor for organic solar cells. *Journal of Materials Chemistry C* **3**, 4698-4705 (2015).
33. A. M. Raynor, A. Gupta, H. Patil, A. Bilic, S. V. Bhosale, A diketopyrrolopyrrole and benzothiadiazole based small molecule electron acceptor: design, synthesis, characterization and photovoltaic properties. *RSC Advances* **4**, 57635-57638 (2014).
34. H. U. Kim *et al.*, High open circuit voltage organic photovoltaic cells fabricated using 9,9'-bifluorenylidene as a non-fullerene type electron acceptor. *Chemical Communications* **49**, 10950-10952 (2013).
35. J. W. Jung, W. H. Jo, Low-Bandgap Small Molecules as Non-Fullerene Electron Acceptors Composed of Benzothiadiazole and Diketopyrrolopyrrole for All Organic Solar Cells. *Chemistry of Materials* **27**, 6038-6043 (2015).
36. Y. Kim, C. E. Song, S.-J. Moon, E. Lim, Rhodanine dye-based small molecule acceptors for organic photovoltaic cells. *Chemical Communications* **50**, 8235-8238 (2014).
37. Y. Zhang *et al.*, Fluorene-centered perylene monoimides as potential non-fullerene acceptor in organic solar cells. *Organic Electronics* **21**, 184-191 (2015).
38. H. Li *et al.*, Fine-Tuning the 3D Structure of Nonfullerene Electron Acceptors Toward High-Performance Polymer Solar Cells. *Advanced Materials* **27**, 3266-3272 (2015).

39. H. Lin *et al.*, High-Performance Non-Fullerene Polymer Solar Cells Based on a Pair of Donor–Acceptor Materials with Complementary Absorption Properties. *Advanced Materials* **27**, 7299-7304 (2015).
40. G. D. Sharma, M. Anil Reddy, D. V. Ramana, M. Chandrasekharam, A novel carbazole-phenothiazine dyad small molecule as a non-fullerene electron acceptor for polymer bulk heterojunction solar cells. *RSC Advances* **4**, 33279-33285 (2014).
41. Y. Lin, Y. Li, X. Zhan, A Solution-Processable Electron Acceptor Based on Dibenzosilole and Diketopyrrolopyrrole for Organic Solar Cells. *Advanced Energy Materials* **3**, 724-728 (2013).
42. J. T. Bloking *et al.*, Solution-Processed Organic Solar Cells with Power Conversion Efficiencies of 2.5% using Benzothiadiazole/Imide-Based Acceptors. *Chemistry of Materials* **23**, 5484-5490 (2011).
43. Y. Zhou *et al.*, Non-fullerene acceptors containing fluoranthene-fused imides for solution-processed inverted organic solar cells. *Chemical Communications* **49**, 5802-5804 (2013).
44. K. N. Winzenberg *et al.*, Indan-1,3-dione electron-acceptor small molecules for solution-processable solar cells: a structure-property correlation. *Chemical Communications* **49**, 6307-6309 (2013).
45. D. Sun *et al.*, Non-Fullerene-Acceptor-Based Bulk-Heterojunction Organic Solar Cells with Efficiency over 7%. *Journal of the American Chemical Society* **137**, 11156-11162 (2015).
46. Y. Kim *et al.*, DPP-based small molecule, non-fullerene acceptors for "channel II" charge generation in OPVs and their improved performance in ternary cells. *RSC Advances* **5**, 4811-4821 (2015).
47. H. Li *et al.*, Beyond Fullerenes: Design of Nonfullerene Acceptors for Efficient Organic Photovoltaics. *Journal of the American Chemical Society* **136**, 14589-14597 (2014).
48. L. Chen *et al.*, A non-fullerene acceptor with all "A" units realizing high open-circuit voltage solution-processed organic photovoltaics. *Journal of Materials Chemistry A* **2**, 2657-2662 (2014).
49. Y. Wu *et al.*, A planar electron acceptor for efficient polymer solar cells. *Energy & Environmental Science* **8**, 3215-3221 (2015).
50. R.-Q. Lu *et al.*, Corannulene derivatives as non-fullerene acceptors in solution-processed bulk heterojunction solar cells. *Journal of Materials Chemistry A* **2**, 20515-20519 (2014).
51. S. Holliday *et al.*, A Rhodanine Flanked Nonfullerene Acceptor for Solution-Processed Organic Photovoltaics. *Journal of the American Chemical Society* **137**, 898-904 (2015).
52. Y. Lin *et al.*, A Twisted Dimeric Perylene Diimide Electron Acceptor for Efficient Organic Solar Cells. *Advanced Energy Materials* **4**, n/a-n/a (2014).
53. A. M. Poe *et al.*, Small molecule BODIPY dyes as non-fullerene acceptors in bulk heterojunction organic photovoltaics. *Chemical Communications* **50**, 2913-2915 (2014).
54. A. Gupta *et al.*, Crowning of dibenzosilole with a naphthalenediimide functional group to prepare an electron acceptor for organic solar cells. *Dyes and Pigments* **120**, 314-321 (2015).
55. M. Schubert *et al.*, Influence of Aggregation on the Performance of All-Polymer Solar Cells Containing Low-Bandgap Naphthalenediimide Copolymers. *Advanced Energy Materials* **2**, 369-380 (2012).
56. P. E. Schwenn *et al.*, A Small Molecule Non-fullerene Electron Acceptor for Organic Solar Cells. *Advanced Energy Materials* **1**, 73-81 (2011).
57. C. B. Nielsen, S. Holliday, H.-Y. Chen, S. J. Cryer, I. McCulloch, Non-Fullerene Electron Acceptors for Use in Organic Solar Cells. *Accounts of Chemical Research* **48**, 2803-2812 (2015).
58. D. P. Ostrowski, U. Koldemir, R. Anderson, A. Sellinger, S. E. Shaheen, in *Photovoltaic Specialist Conference (PVSC), 2014 IEEE 40th*. (2014), pp. 1-3.
59. L. J. Richter *et al.*, In Situ Morphology Studies of the Mechanism for Solution Additive Effects on the Formation of Bulk Heterojunction Films. *Advanced Energy Materials* **5**, n/a-n/a (2015).

60. J. W. Jung, F. Liu, T. P. Russell, W. H. Jo, A high mobility conjugated polymer based on dithienothiophene and diketopyrrolopyrrole for organic photovoltaics. *Energy & Environmental Science* **5**, 6857-6861 (2012).
61. K. Kawashima, Y. Tamai, H. Ohkita, I. Osaka, K. Takimiya, High-efficiency polymer solar cells with small photon energy loss. *Nat Commun* **6**, (2015).
62. G. Wei *et al.*, Functionalized Squaraine Donors for Nanocrystalline Organic Photovoltaics. *ACS Nano* **6**, 972-978 (2012).
63. C. B. Nielsen *et al.*, Random benzotrithiophene-based donor-acceptor copolymers for efficient organic photovoltaic devices. *Chemical Communications* **48**, 5832-5834 (2012).
64. J. W. Jung, F. Liu, T. P. Russell, W. H. Jo, Semi-crystalline random conjugated copolymers with panchromatic absorption for highly efficient polymer solar cells. *Energy & Environmental Science* **6**, 3301-3307 (2013).
65. M. Wang *et al.*, High Open Circuit Voltage in Regioregular Narrow Band Gap Polymer Solar Cells. *Journal of the American Chemical Society* **136**, 12576-12579 (2014).
66. C.-C. Chen *et al.*, High-performance semi-transparent polymer solar cells possessing tandem structures. *Energy & Environmental Science* **6**, 2714-2720 (2013).
67. J. You *et al.*, A polymer tandem solar cell with 10.6% power conversion efficiency. *Nat Commun* **4**, 1446 (2013).
68. K. H. Hendriks, G. H. L. Heintges, V. S. Gevaerts, M. M. Wienk, R. A. J. Janssen, High-Molecular-Weight Regular Alternating Diketopyrrolopyrrole-based Terpolymers for Efficient Organic Solar Cells. *Angewandte Chemie International Edition* **52**, 8341-8344 (2013).
69. H. Choi *et al.*, Small-Bandgap Polymer Solar Cells with Unprecedented Short-Circuit Current Density and High Fill Factor. *Advanced Materials* **27**, 3318-3324 (2015).
70. K. Vandewal *et al.*, Quantification of Quantum Efficiency and Energy Losses in Low Bandgap Polymer:Fullerene Solar Cells with High Open-Circuit Voltage. *Advanced Functional Materials* **22**, 3480-3490 (2012).
71. B. Kan *et al.*, A Series of Simple Oligomer-like Small Molecules Based on Oligothiophenes for Solution-Processed Solar Cells with High Efficiency. *Journal of the American Chemical Society* **137**, 3886-3893 (2015).
72. L. Dou *et al.*, Synthesis of 5H-Dithieno[3,2-b:2′,3′-d]pyran as an Electron-Rich Building Block for Donor–Acceptor Type Low-Bandgap Polymers. *Macromolecules* **46**, 3384-3390 (2013).
73. D. H. Wang, A. K. K. Kyaw, J.-R. Pouliot, M. Leclerc, A. J. Heeger, Enhanced Power Conversion Efficiency of Low Band-Gap Polymer Solar Cells by Insertion of Optimized Binary Processing Additives. *Advanced Energy Materials* **4**, n/a-n/a (2014).
74. Z. Ma, E. Wang, K. Vandewal, M. R. Andersson, F. Zhang, Enhance performance of organic solar cells based on an isoindigo-based copolymer by balancing absorption and miscibility of electron acceptor. *Applied Physics Letters* **99**, 143302 (2011).
75. E. H. Jung, W. H. Jo, π-Extended low bandgap polymer based on isoindigo and thienylvinylene for high performance polymer solar cells. *Energy & Environmental Science* **7**, 650-654 (2014).
76. W. Li, A. Furlan, K. H. Hendriks, M. M. Wienk, R. A. J. Janssen, Efficient Tandem and Triple-Junction Polymer Solar Cells. *Journal of the American Chemical Society* **135**, 5529-5532 (2013).
77. Z. Zeng, Y. Li, J. Deng, Q. Huang, Q. Peng, Synthesis and photovoltaic performance of low band gap copolymers based on diketopyrrolopyrrole and tetrathienoacene with different conjugated bridges. *Journal of Materials Chemistry A* **2**, 653-662 (2014).
78. L. Ye, S. Zhang, W. Zhao, H. Yao, J. Hou, Highly Efficient 2D-Conjugated Benzodithiophene-Based Photovoltaic Polymer with Linear Alkylthio Side Chain. *Chemistry of Materials* **26**, 3603-3605 (2014).

79. E. Wang *et al.*, An isoindigo-based low band gap polymer for efficient polymer solar cells with high photo-voltage. *Chemical Communications* **47**, 4908-4910 (2011).
80. S. Zhang *et al.*, Enhanced Photovoltaic Performance of Diketopyrrolopyrrole (DPP)-Based Polymers with Extended π Conjugation. *The Journal of Physical Chemistry C* **117**, 9550-9557 (2013).
81. C. Cui, W.-Y. Wong, Y. Li, Improvement of open-circuit voltage and photovoltaic properties of 2D-conjugated polymers by alkylthio substitution. *Energy & Environmental Science* **7**, 2276-2284 (2014).
82. R. Fitzner *et al.*, Correlation of π-Conjugated Oligomer Structure with Film Morphology and Organic Solar Cell Performance. *Journal of the American Chemical Society* **134**, 11064-11067 (2012).
83. Y. Sun *et al.*, Solution-processed small-molecule solar cells with 6.7% efficiency. *Nat Mater* **11**, 44-48 (2012).
84. Q. Zhang *et al.*, A solution-processed high performance organic solar cell using a small molecule with the thieno[3,2-b]thiophene central unit. *Chemical Communications* **51**, 15268-15271 (2015).
85. P. Zhou, Z.-G. Zhang, Y. Li, X. Chen, J. Qin, Thiophene-Fused Benzothiadiazole: A Strong Electron-Acceptor Unit to Build D–A Copolymer for Highly Efficient Polymer Solar Cells. *Chemistry of Materials* **26**, 3495-3501 (2014).
86. A. T. Yiu *et al.*, Side-Chain Tunability of Furan-Containing Low-Band-Gap Polymers Provides Control of Structural Order in Efficient Solar Cells. *Journal of the American Chemical Society* **134**, 2180-2185 (2012).
87. E. Zhou *et al.*, Diketopyrrolopyrrole-Based Semiconducting Polymer for Photovoltaic Device with Photocurrent Response Wavelengths up to 1.1 µm. *Macromolecules* **43**, 821-826 (2010).
88. W. Li *et al.*, Universal Correlation between Fibril Width and Quantum Efficiency in Diketopyrrolopyrrole-Based Polymer Solar Cells. *Journal of the American Chemical Society* **135**, 18942-18948 (2013).
89. Y. Liu *et al.*, Aggregation and morphology control enables multiple cases of high-efficiency polymer solar cells. *Nat Commun* **5**, (2014).
90. F. Huang *et al.*, Development of New Conjugated Polymers with Donor−π-Bridge−Acceptor Side Chains for High Performance Solar Cells. *Journal of the American Chemical Society* **131**, 13886-13887 (2009).
91. S. Zhang *et al.*, Side Chain Selection for Designing Highly Efficient Photovoltaic Polymers with 2D-Conjugated Structure. *Macromolecules* **47**, 4653-4659 (2014).
92. Y. Kim, H. R. Yeom, J. Y. Kim, C. Yang, High-efficiency polymer solar cells with a cost-effective quinoxaline polymer through nanoscale morphology control induced by practical processing additives. *Energy & Environmental Science* **6**, 1909-1916 (2013).
93. J. J. Intemann *et al.*, Highly Efficient Inverted Organic Solar Cells Through Material and Interfacial Engineering of Indacenodithieno[3,2-b]thiophene-Based Polymers and Devices. *Advanced Functional Materials* **24**, 1465-1473 (2014).
94. E. Wang *et al.*, An Easily Accessible Isoindigo-Based Polymer for High-Performance Polymer Solar Cells. *Journal of the American Chemical Society* **133**, 14244-14247 (2011).
95. R. C. Coffin, J. Peet, J. Rogers, G. C. Bazan, Streamlined microwave-assisted preparation of narrow-bandgap conjugated polymers for high-performance bulk heterojunction solar cells. *Nat Chem* **1**, 657-661 (2009).
96. J. Peet *et al.*, Efficiency enhancement in low-bandgap polymer solar cells by processing with alkane dithiols. *Nat Mater* **6**, 497-500 (2007).

97. J. You *et al.*, 10.2% Power Conversion Efficiency Polymer Tandem Solar Cells Consisting of Two Identical Sub-Cells. *Advanced Materials* **25**, 3973-3978 (2013).
98. M. Zhang, X. Guo, W. Ma, H. Ade, J. Hou, A Large-Bandgap Conjugated Polymer for Versatile Photovoltaic Applications with High Performance. *Advanced Materials* **27**, 4655-4660 (2015).
99. B. Kan *et al.*, Solution-Processed Organic Solar Cells Based on Dialkylthiol-Substituted Benzodithiophene Unit with Efficiency near 10%. *Journal of the American Chemical Society* **136**, 15529-15532 (2014).
100. C. E. Small *et al.*, High-efficiency inverted dithienogermole-thienopyrrolodione-based polymer solar cells. *Nat Photon* **6**, 115-120 (2012).
101. I. Meager *et al.*, Photocurrent Enhancement from Diketopyrrolopyrrole Polymer Solar Cells through Alkyl-Chain Branching Point Manipulation. *Journal of the American Chemical Society* **135**, 11537-11540 (2013).
102. J. Yuan *et al.*, Efficient Polymer Solar Cells with a High Open Circuit Voltage of 1 Volt. *Advanced Functional Materials* **23**, 885-892 (2013).
103. V. Vohra *et al.*, Efficient inverted polymer solar cells employing favourable molecular orientation. *Nat Photon* **9**, 403-408 (2015).
104. I. Osaka, T. Kakara, N. Takemura, T. Koganezawa, K. Takimiya, Naphthodithiophene–Naphthobisthiadiazole Copolymers for Solar Cells: Alkylation Drives the Polymer Backbone Flat and Promotes Efficiency. *Journal of the American Chemical Society* **135**, 8834-8837 (2013).
105. L. Yuan *et al.*, Oligomeric Donor Material for High-Efficiency Organic Solar Cells: Breaking Down a Polymer. *Advanced Materials* **27**, 4229-4233 (2015).
106. K. H. Hendriks, W. Li, M. M. Wienk, R. A. J. Janssen, Band Gap Control in Diketopyrrolopyrrole-Based Polymer Solar Cells Using Electron Donating Side Chains. *Advanced Energy Materials* **3**, 674-679 (2013).
107. V. S. Gevaerts, A. Furlan, M. M. Wienk, M. Turbiez, R. A. J. Janssen, Solution Processed Polymer Tandem Solar Cell Using Efficient Small and Wide bandgap Polymer:Fullerene Blends. *Advanced Materials* **24**, 2130-2134 (2012).
108. W. Yue *et al.*, A Thieno[3,2-b][1]benzothiophene Isoindigo Building Block for Additive- and Annealing-Free High-Performance Polymer Solar Cells. *Advanced Materials* **27**, 4702-4707 (2015).
109. T. L. Nguyen *et al.*, Semi-crystalline photovoltaic polymers with efficiency exceeding 9% in a ~300 nm thick conventional single-cell device. *Energy & Environmental Science* **7**, 3040-3051 (2014).
110. Y. Liang *et al.*, For the Bright Future—Bulk Heterojunction Polymer Solar Cells with Power Conversion Efficiency of 7.4%. *Advanced Materials* **22**, E135-E138 (2010).
111. C. Cabanetos *et al.*, Linear Side Chains in Benzo[1,2-b:4,5-b']dithiophene–Thieno[3,4-c]pyrrole-4,6-dione Polymers Direct Self-Assembly and Solar Cell Performance. *Journal of the American Chemical Society* **135**, 4656-4659 (2013).
112. K. Li *et al.*, Development of Large Band-Gap Conjugated Copolymers for Efficient Regular Single and Tandem Organic Solar Cells. *Journal of the American Chemical Society* **135**, 13549-13557 (2013).
113. T.-Y. Chu *et al.*, Bulk Heterojunction Solar Cells Using Thieno[3,4-c]pyrrole-4,6-dione and Dithieno[3,2-b:2′,3′-d]silole Copolymer with a Power Conversion Efficiency of 7.3%. *Journal of the American Chemical Society* **133**, 4250-4253 (2011).
114. K. Sun *et al.*, A molecular nematic liquid crystalline material for high-performance organic photovoltaics. *Nat Commun* **6**, (2015).
115. X. Guo *et al.*, Polymer solar cells with enhanced fill factors. *Nat Photon* **7**, 825-833 (2013).

116. D. Fujishima *et al.*, Organic thin-film solar cell employing a novel electron-donor material. *Solar Energy Materials and Solar Cells* **93**, 1029-1032 (2009).
117. J. Cao *et al.*, A pentacyclic aromatic lactam building block for efficient polymer solar cells. *Energy & Environmental Science* **6**, 3224-3228 (2013).
118. N. S. Baek *et al.*, High Performance Amorphous Metallated π-Conjugated Polymers for Field-Effect Transistors and Polymer Solar Cells. *Chemistry of Materials* **20**, 5734-5736 (2008).
119. W. Yue *et al.*, Novel NIR-absorbing conjugated polymers for efficient polymer solar cells: effect of alkyl chain length on device performance. *Journal of Materials Chemistry* **19**, 2199-2206 (2009).
120. W. Li, W. S. C. Roelofs, M. M. Wienk, R. A. J. Janssen, Enhancing the Photocurrent in Diketopyrrolopyrrole-Based Polymer Solar Cells via Energy Level Control. *Journal of the American Chemical Society* **134**, 13787-13795 (2012).
121. Y. He, H.-Y. Chen, J. Hou, Y. Li, Indene−C60 Bisadduct: A New Acceptor for High-Performance Polymer Solar Cells. *Journal of the American Chemical Society* **132**, 1377-1382 (2010).
122. F. Yang, K. Sun, S. R. Forrest, Efficient Solar Cells Using All-Organic Nanocrystalline Networks. *Advanced Materials* **19**, 4166-4171 (2007).
123. E. Voroshazi *et al.*, Novel bis-C60 derivative compared to other fullerene bis-adducts in high efficiency polymer photovoltaic cells. *Journal of Materials Chemistry* **21**, 17345-17352 (2011).
124. L. J. Lindgren *et al.*, Synthesis, Characterization, and Devices of a Series of Alternating Copolymers for Solar Cells. *Chemistry of Materials* **21**, 3491-3502 (2009).
125. T. Ameri *et al.*, Morphology analysis of near IR sensitized polymer/fullerene organic solar cells by implementing low bandgap heteroanalogue C-/Si-PCPDTBT. *Journal of Materials Chemistry A* **2**, 19461-19472 (2014).
126. T. W. Holcombe, C. H. Woo, D. F. J. Kavulak, B. C. Thompson, J. M. J. Fréchet, All-Polymer Photovoltaic Devices of Poly(3-(4-n-octyl)-phenylthiophene) from Grignard Metathesis (GRIM) Polymerization. *Journal of the American Chemical Society* **131**, 14160-14161 (2009).
127. Y. Zhang *et al.*, Synthesis and photovoltaic properties of an n-type two-dimension-conjugated polymer based on perylene diimide and benzodithiophene with thiophene conjugated side chains. *Journal of Materials Chemistry A* **3**, 18442-18449 (2015).
128. C. Mu *et al.*, High-Efficiency All-Polymer Solar Cells Based on a Pair of Crystalline Low-Bandgap Polymers. *Advanced Materials* **26**, 7224-7230 (2014).
129. Y. Liu *et al.*, All polymer photovoltaics: From small inverted devices to large roll-to-roll coated and printed solar cells. *Solar Energy Materials and Solar Cells* **112**, 157-162 (2013).
130. T. Kietzke, H.-H. Hörhold, D. Neher, Efficient Polymer Solar Cells Based on M3EH−PPV. *Chemistry of Materials* **17**, 6532-6537 (2005).
131. T. Earmme, Y.-J. Hwang, N. M. Murari, S. Subramaniyan, S. A. Jenekhe, All-Polymer Solar Cells with 3.3% Efficiency Based on Naphthalene Diimide-Selenophene Copolymer Acceptor. *Journal of the American Chemical Society* **135**, 14960-14963 (2013).
132. E. Zhou *et al.*, All-Polymer Solar Cells from Perylene Diimide Based Copolymers: Material Design and Phase Separation Control. *Angewandte Chemie International Edition* **50**, 2799-2803 (2011).
133. E. Zhou, J. Cong, K. Hashimoto, K. Tajima, Control of Miscibility and Aggregation Via the Material Design and Coating Process for High-Performance Polymer Blend Solar Cells. *Advanced Materials* **25**, 6991-6996 (2013).
134. E. Zhou *et al.*, Synthesis and application of poly(fluorene-alt-naphthalene diimide) as an n-type polymer for all-polymer solar cells. *Chemical Communications* **48**, 5283-5285 (2012).
